# Seasonal Differences of Gene Expression Profiles in Song Sparrow (*Melospiza melodia*) Hypothalamus in Relation to Territorial Aggression

**DOI:** 10.1371/journal.pone.0008182

**Published:** 2009-12-04

**Authors:** Motoko Mukai, Kirstin Replogle, Jenny Drnevich, Gang Wang, Douglas Wacker, Mark Band, David F. Clayton, John C. Wingfield

**Affiliations:** 1 Department of Neurobiology, Physiology, and Behavior, University of California Davis, Davis, California, United States of America; 2 Department of Veterinary Biosciences, University of Illinois, Champaign, Illinois, United States of America; 3 Department of Cell and Developmental Biology, University of Illinois, Champaign, Illinois, United States of America; 4 Institute for Genomic Biology, University of Illinois, Champaign, Illinois, United States of America; 5 Neuroscience Program, University of Illinois, Champaign, Illinois, United States of America; 6 W.M. Keck Center for Comparative and Functional Genomics, University of Illinois at Urbana-Champaign, Urbana, Illinois, United States of America; 7 Department of Biology, University of Washington, Seattle, Washington, United States of America; University of Parma, Italy

## Abstract

**Background:**

Male song sparrows (*Melospiza melodia*) are territorial year-round; however, neuroendocrine responses to simulated territorial intrusion (STI) differ between breeding (spring) and non-breeding seasons (autumn). In spring, exposure to STI leads to increases in luteinizing hormone and testosterone, but not in autumn. These observations suggest that there are fundamental differences in the mechanisms driving neuroendocrine responses to STI between seasons. Microarrays, spotted with EST cDNA clones of zebra finch, were used to explore gene expression profiles in the hypothalamus after territorial aggression in two different seasons.

**Methodology/Principal Findings:**

Free-living territorial male song sparrows were exposed to either conspecific or heterospecific (control) males in an STI in spring and autumn. Behavioral data were recorded, whole hypothalami were collected, and microarray hybridizations were performed. Quantitative PCR was performed for validation. Our results show 262 cDNAs were differentially expressed between spring and autumn in the control birds. There were 173 cDNAs significantly affected by STI in autumn; however, only 67 were significantly affected by STI in spring. There were 88 cDNAs that showed significant interactions in both season and STI.

**Conclusions/Significance:**

Results suggest that STI drives differential genomic responses in the hypothalamus in the spring vs. autumn. The number of cDNAs differentially expressed in relation to season was greater than in relation to social interactions, suggesting major underlying seasonal effects in the hypothalamus which may determine the differential response upon social interaction. Functional pathway analyses implicated genes that regulate thyroid hormone action and neuroplasticity as targets of this neuroendocrine regulation.

## Introduction

Testosterone (T) has classically been regarded as a major factor in the control of vertebrate aggression, at least in reproductive contexts. However, regulation of aggression may be far more complex as T manipulations have had variable results on aggressive behaviors in different species [1], [2], [3]. Moreover, T secretion upon social interaction has been observed in some but not all species (reviewed in [4]), further adding to the complexity of T's role in natural aggression. The “challenge hypothesis” [5] attempts to explain the variable linkage of T and aggression during the breeding season. It suggests that a correlation between the two exists only during social instability, such as during establishment of dominance relationships and/or territorial boundaries, or when animals are “challenged” by a conspecific male for their territories and/or mates. The challenge hypothesis has been supported by studies in over 85 avian species [5], [6], [7] as well as in over 150 other vertebrates such as fish [8], reptiles [9], [10] and mammals [11] including humans [12].

Male song sparrows, *Melospiza melodia*, have been one of the ideal models for testing the challenge hypothesis and exploring the underlying mechanisms [5]. The influence of social interaction on testosterone levels is clearly depicted by a difference in patterns of systemic testosterone levels in captive vs. free-living song sparrows, the latter more likely to be socially instable or be challenged [reviewed in [13]]. Sedentary populations that reside in western Washington State, *Melospiza melodia morphna*, maintain year-round aggression towards conspecific male territorial intruders, except for brief period during molt when males show little or no aggression.

However, there is a difference in their responses to territorial challenge between seasons at the neuroendocrine level. During the breeding season, circulating levels of luteinizing hormone (LH) and testosterone (T) are high, and they increase to even higher levels upon social interaction [14], [15]. In the non-breeding season, gonads are regressed, circulating LH and T levels are undetectable, and remain so even after social interactions [16]. Since males show behavioral aggression even in the non-breeding season, an increase of systemic T upon social interaction must not be necessary to activate aggression in this species; although it may increase the intensity and persistence of the behavior [17].

Emerging evidence has suggested locally synthesized but not systemic T and estrogen in the brain play a major role in triggering aggression in song sparrows during the non-breeding season [18], [19]. The sensitivity of the brain to these hormones may also increase during the non-breeding season. For example, mRNA up-regulations of their receptors, *ERα* and *AR*, during non-breeding seasons has been reported in spotted antbirds (*Hylophylax n. naevioides*), another species that shows year-round aggression [20].

Release of gonadotropin-releasing hormone (GnRH) at the hypothalamus will usually lead to a subsequent increase and release of LH from the pituitary, which will then stimulate the Leydig cells in the testis to increase T systemically. Recently, our laboratory found that FOS (also known as c-fos) protein is differentially expressed in the hypothalamus after territorial intrusion in breeding vs. non-breeding season. This seasonal difference was observed in brain nuclei that play a role in neuroendocrine response, such as the *median eminence* (site of GnRH release). Territorial intrusion also induced FOS in other brain areas that play a role in memory, emotion and behavior including aggression, but with no differential effect of season [K. Soma, S. Meddle and J. Wingfield unpublished data]. Expression of the immediate-early response gene, *FOS*, is commonly used as an indicator of functional activation of cells in a particular brain region. Therefore, these findings imply that brain regions important for the neuroendocrine response may be selectively activated by territorial aggression, or at least activated in a different way, during breeding season. However, the detailed mechanism driving this seasonal difference in neuroendocrine response remains unknown.

The main aim of this study was to explore and understand the mechanisms underlying seasonal differences in the neuroendocrine response upon social interaction. Basal gene expression was also compared between two different seasons, breeding and non-breeding, to discern the differences of expression status without the territorial challenge. We collected hypothalami from free-living male song sparrows (*Melospiza melodia morphna*) in both breeding and non-breeding seasons after eliciting territorial aggression in the field by challenge experiments, called simulated territorial intrusions (STI), and we analyzed gene expression using microarray resources of the Songbird Neurogenomics Initiative (SoNG) [21].

Our results show that territorial aggression is associated with hypothalamic gene responses, but the profile clearly differs between the breeding and non-breeding season. We found that cDNAs that code genes that regulate thyroid hormone and neuroplasticity are differentially expressed across seasons with or without territorial intrusion which may contribute to the regulation of GnRH release. Somewhat surprisingly, fewer genes responded to territorial challenge in the breeding season compared to non-breeding season. This suggests the existence of a refined pathway to quickly respond to territorial intrusions with further increases in testosterone under challenged conditions in the breeding season, and points to a possible mechanism underlying the challenge hypothesis.

## Results

### Territorial Behavior

**Figure 1:**
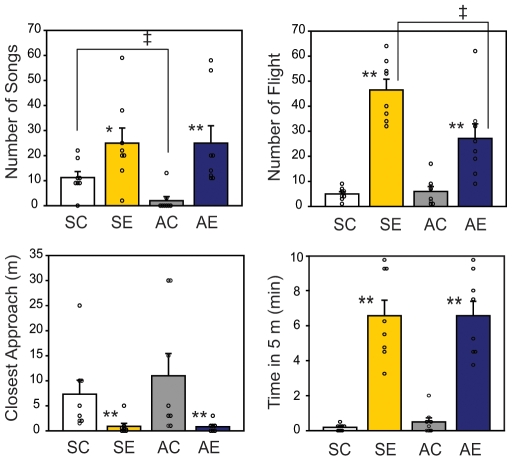
Aggressive behavior of sparrows subjected to simulated territorial intrusion during breeding and non-breeding season. Male song sparrows (n = 8/group) in their territory were exposed to a caged conspecific male decoy in addition to a recorded male song playbacks in breeding season, spring (SE) and non-breeding season, autumn (AE). As controls (AC, SC), a white-crowned sparrow decoy and recorded songs were used. Behavior of a subject was recorded for number of songs, number of flight, closest approach to the decoy (meters; m), and time spend within 5-m of the decoy. Kruskal-Wallis 1-way ANOVA followed by Mann-Whitney U test; significant at * p<0.05, ** p<0.01 between experimental conditions of the same season, or at † p<0.05 between seasons under same experimental conditions. Results are shown in mean±SEM.

Simulated territorial intrusion experiments (STI) were carried out in the field in western Washington in breeding (spring) and non-breeding seasons (autumn to winter; called autumn herein). To quantify the territorial behavior of the birds upon STI, 4 behavioral endpoints were recorded: number of full songs (a characteristic territorial vocalization), number of flights, closest approach (meters; m) to the decoy, and time spent within 5-m of the decoy. Behavioral data obtained from birds during an STI or control were consistent with previous reports [16]. Birds responded with increased aggression towards a conspecific intruder both in spring and autumn (SE and AE) compared to controls (SC and AC, respectively; Fig. 1). In spring, an STI increased the number of songs by 2.2-fold (SE, 25±6.0 songs; SC, 11.3±2.4 songs; U_(8,8)_ = 52.5, p<0.05) and the number of flights by 9.3-fold (SE, 46.5±4.3 flights; SC, 5±0.9 flights; U_(8,8)_ = 64, p<0.01), compared to controls. In autumn, there was decrease in the basal number of songs compared to spring (AC; 2±1.6 songs; U_(8,8)_ = 54, p<0.05 vs. SC) but birds still responded robustly to STI with a 12.5 fold increase (AE; 25±6.9 songs) compared to controls (U_(8,8)_ = 61, p<0.01, AE vs. AC). STI also increased the number of flights in autumn (AE, 27.1±5.8 flights; AC, 6±1.9 flights; U_(8,8)_ = 61.5, p<0.01); although, it did not reach the same level as in the spring. Distance of closest approach to the decoy was decreased in both seasons (U_(8,8)_ = 59.5 for spring; U_(8,8)_ = 58 for autumn; p<0.01) with STI (0.9±0.6 and 0.8±0.4 m; SE and AE, respectively) compared to controls of the same season (7.3±2.8 and 11±4.4 m; SC and AC, respectively). Time spent within 5-m of the decoy was increased in both seasons (U_(8,8)_ = 64, p<0.01) with STI (6.6±0.9 and 6.6±0.8 min; SE and AE, respectively) compared to controls of the same season (0.2±0.1 and 0.5±0.2 min; SC and AC).

### Identification of Differentially Expressed Genes by Microarray

To identify genes that are differentially expressed during territorial aggression in breeding and non-breeding season, bird hypothalami (with *pars tuberalis* of pituitary) were collected, RNA extracted and labeled-cDNA samples were hybridized to a custom-made cDNA microarray. This array was constructed with cDNAs derived from zebra finch brain tissues, which had been validated for use with various songbird species including song sparrows by comparative genomic hybridization [21]. The detailed description of this array platform is described previously [21] but briefly, this spotted cDNA microarray represents 17,214 non-redundant products of an estimated 11,500–15,000 genes. Some genes were represented by several cDNA clones from different parts of the transcript; in these cases, the different cDNAs may measure different mRNA isoforms from the same gene (e.g. variations in splicing or start site) having different regulation patterns. Therefore the initial statistical analyses treated each cDNA spot independently. Each array was hybridized with the sample from a single song sparrow along with SoNG's universal reference derived from zebra finch brain, for total of 31 hybridizations.

**Figure 2:**
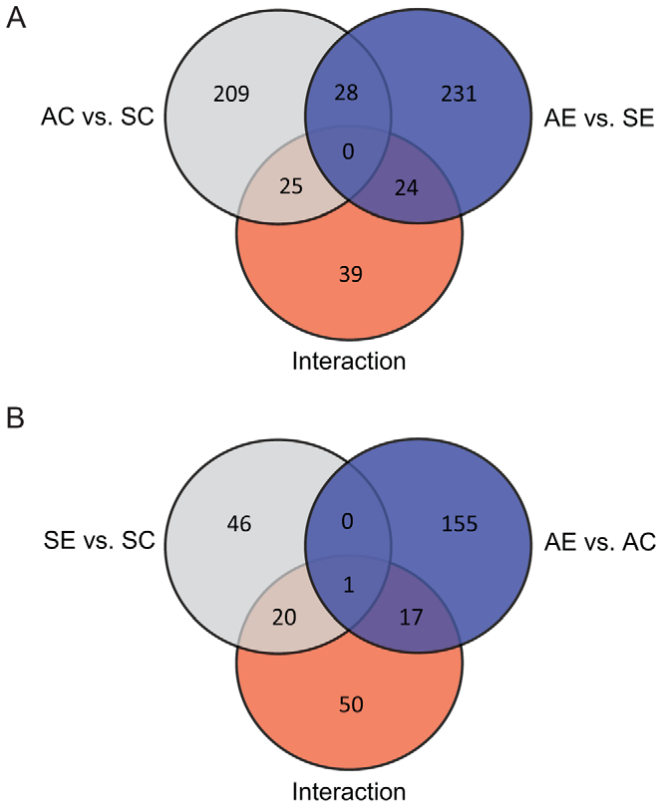
Venn-diagram of regulated cDNA spots in different seasons and with simulated territorial intrusions. A) Number of regulated cDNA spots in autumn compared to spring in either control or STI groups (AC vs. SC, 262 total; AE vs. SE, 283 total), and those that showed interaction effect of season and STI (88 total). B) Number of regulated cDNA spots in STI compared to controls in each season (SE vs. SC, 67 total; AE vs. AC, 173 total), and those that showed interaction effect of season and STI. The numbers within the overlapped circle indicates an overlap of differentially expressed cDNAs. Cell-means model was used for statistics, significant at p<0.01.

A total of 727 cDNA spots showed significance at p<0.01 for AC vs. SC, AE vs. SE, AE vs. AC, SE vs. SC, AE vs. SE, or interaction (by cell-means model followed by individual posthoc tests, see *Methods* section for details). The Venn-diagram (Fig. 2) shows the number of significant cDNA spots in AC vs. SC, AE vs. AE, and interaction (Fig. 2A); and SE vs. SC, AE v. AC, and interaction (Fig. 2B). For example, 262 cDNAs were differentially expressed between spring and autumn in birds not exposed to STI (AC vs. SC; Fig. 2A, p<0.01). Of those, 115 were down-regulated and 147 were up-regulated in autumn compared to spring (Table S1). There was also a seasonal effect in the birds that had been exposed to STI (283 cDNA spots total, Fig. 2A; 143 down-regulated and 140 up-regulated in AE vs. SE; Table S2). There were 88 cDNAs that were significant (p<0.01) in the interaction of season and STI (Fig. 2B, Table S5). Among seasonally affected cDNAs, only 25 of the AC vs. SC, and only 24 of the AE vs. SE overlapped with those affected in the interaction (Fig. 2A). STI had an effect on expression in both seasons, although the magnitude of the effect was different in spring and autumn. In spring, 67 cDNAs were significantly different between control and STI birds in spring (Fig. 2B). Of those, 31 cDNAs were down-regulated and 36 were up-regulated with STI (Table S3). In autumn, 173 cDNAs were significantly different between control and STI birds (Fig. 2B, p<0.01). Of those, 77 cDNAs were down-regulated and 96 were up-regulated with STI (Table S4). Only one cDNA, annotated as forming binding protein 1-like (*FNBP1L*), was differentially regulated by STI in both spring and autumn (Fig. 2B), although it was regulated in the opposite direction (up in spring and down in autumn). Among cDNAs affected in the interaction, 21 cDNA clones overlapped with SE vs. SC significant list, 18 cDNAs overlapped with AE vs. AC significant list (Fig. 2B).

**Figure 3:**
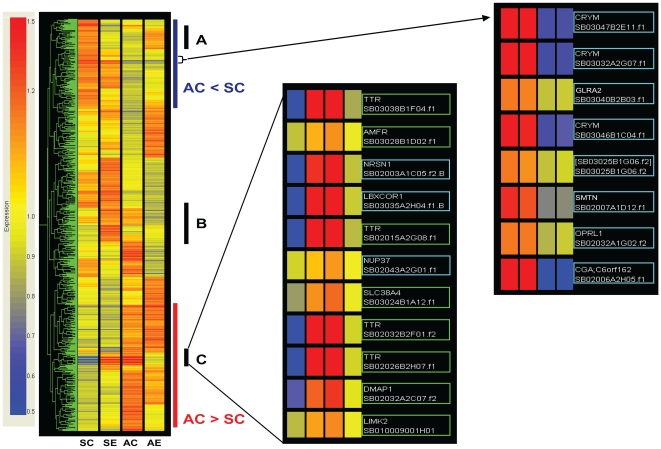
Hierarchical clustering of all differentially expressed cDNA spots. All cDNA spots that passed the cut-off (p<0.01) in the cell-means model (total of 727 genes) were clustered using Pearson correlation and shown as a heatmap. Up-regulated cDNAs are shown in red, down-regulated cDNAs are shown in blue compared to a mean of all four groups. Examples of two distinct groups are seen in AC vs. SC comparison (blue and red line; down- and up-regulation, respectively). Those cDNA spots affected by interactions are also shown as sections (A, B, C). Portion of AC<SC and section C of the interaction are magnified as a representation of cDNA spots (middle and right panel). Those cDNAs highlighted made cutoff in each comparisons (squared in green with interaction effect, blue with AC vs. SC). Clustering of different cDNA spots of the same gene (*TTR* and *CRYM*) shows consistency of expression patterns among these cDNAs.

Hierarchical cluster analysis of all of the 727 significantly affected cDNA clone spots (Tables S1, S2, S3, S4, and S5) showed various combinations of gene regulation in the different seasons and by social interactions (Fig. 3). Where separate cDNA clones were derived from the same gene, the Pearson correlation analysis often clustered them together supporting the general reliability of our array (e.g. 4 *TTR* cDNAs in the middle panel and 3 *CRYM* cDNAs in the right panel of Fig. 3).

**Figure 4:**
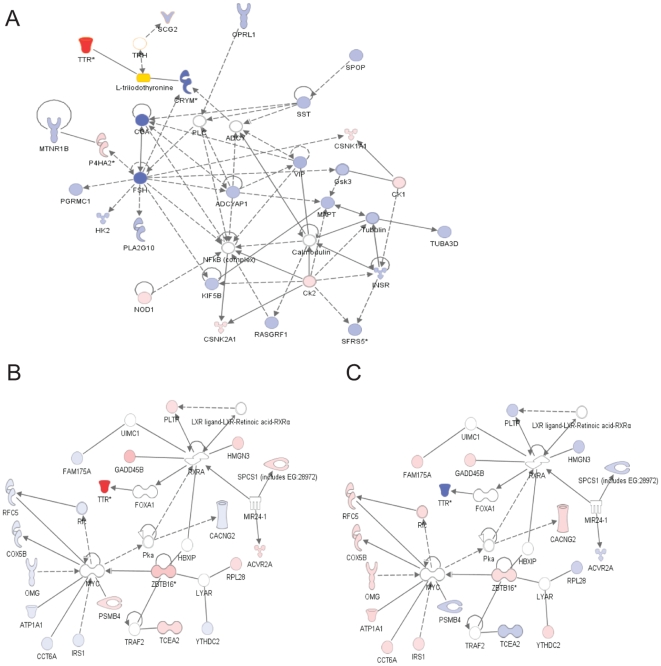
Representative gene network discovered by Ingenuity Pathway Analysis. A) A network affected in AC vs. SC comparison. Red represents up-regulation of the gene in autumn control (AC) vs. spring control (SC), whereas blue represents down-regulation. Color intensity correlates with a degree of fold change. Thyroid hormone (L-triiodothyronine, T_3_) is highlighted by yellow. White represents genes that were either not differentially expressed, not been annotated, or not in the custom-made array. B&C) A network affected by interaction (season×STI). The same network shown with expression fold-changes for SE vs. SC (B) or AE vs. AC (C). Red represents up-regulation of the gene in STIs (SE in B, AE in C) when compared with controls (SC in B, AC in C), whereas blue represents down-regulation. Same as (A) for the white. When there were multiple cDNA clone spots per gene, fold change values that were of most significance were selected.

To identify affected gene networks and functional pathways, pathway analysis was performed using IPA 6 (Ingenuity Systems, Redwood City, CA). Several of prominently affected gene networks involved genes associated with thyroid hormone action and neuroplasticity. We focused on these networks because of their relationship to the control of GnRH secretion. We also saw evidence of coordinated regulation of these networks in the AC vs. SC and in the interaction (representative networks shown in Fig. 4). Table 1 shows representative cDNAs that were affected in these networks, and will be mentioned in the discussion. The complete list is available as Tables S1, S2, S3, S4, and S5.

**Table 1:** Representative cDNAs affected in each comparison.

Spot ID	Gene Symbol	Description	AC vs. SC	AE vs. SE	SE vs. SC	AE vs. AC	Int. Spring	Int. Autumn
**1. Thyroid hormone regulations**								
SB03044B2G04.f1	APP	Amyloid beta (A4) precursor protein				1.2		
**SB02006A2H05.f1**	**CGA**	**Glycoprotein hormones alpha chain precursor**	**−16 (−42.8)**	**−7.4 (−8.3)**				
SB03032A2G07.f1	CRYM	Mu-crystallin homolog	−2.8	−2.4				
SB03046B1C04.f1	CRYM	Mu-crystallin homolog	−2.7	−2.1				
**SB03047B2E11.f1**	**CRYM**	**Mu-crystallin homolog**	**−2.6**	**−2.3**				
SB03048B2D08.f1	HMGN3	High mobility group nucleosomal binding domain 3					1.3	−1.3
SB03037B2H05.f1	HSPA2	Heat shock 70 kDa protein 2	1.8	2				
**SB03044A2D03.f1**	**HSPA2**	**Heat shock 70 kDa protein 2**	**1.6**	**1.8 (2.4)**				
SB02038B2G02.f1	TTR	Transthyretin precursor				−1.2		
**SB02015A2G08.f1**	**TTR**	**Transthyretin precursor**	**13.3**	**(−11)**	**(18.1)**		**7 (18.1)**	**−3.6 (−2.1)**
SB02026B2H07.f1	TTR	Transthyretin precursor	10				6.3	−2.8
SB02032B2F01.f2	TTR	Transthyretin precursor	9.8				5.9	−2.9
SB03038B1F04.f1	TTR	Transthyretin precursor	8.3				5.4	−3
**SB03042A1D05.f1**	**VIP**	**Vasoactive intestinal peptide precursor**	**−1.5**					
**2. Neuroplasticity and transcriptional regulations**								
SB02032A2C07.f2	DMAP1	DNA methyltransferase 1 associated protein 1	1.9				1.7	−1.4
SB03005A2E08.f1	DNALI1	Dynein, axonemal, light intermediate chain 1	1.2					
SB02023A1D07.f1	DYNLL1	Dynein, cytoplasmic, light chain 1	1.3					
**SB02022A2D01.f1**	**GADD45B**	**Growth arrest and DNA-damage-inducible, beta**		**−2.3 (−2.3)**	**2.2 (3.0)**		**2.2 (3.0)**	**1.2 (2.0)**
SB02004A2D01.f1	MRPL12	Mitochondrial ribosomal protein L12	1.3					
SB02023A2A01.f1	MRPS17	Mitochondrial ribosomal protein S17				1.2		
**SB02003A1C05.f2**	**NRSN1**	**Neurensin 1**	**4.2**					
SB03038A2C12.f1	PAPD1	PAP associated domain containing 1				1.2		
SB02016A2E02.f1	RPL3	Ribosomal protein L3 (RPL3)				1.2		
SB02010B1D10.f1	RPS6KA2	Ribosomal protein S6 kinase, polypeptide 2	1.4			−1.3		
SB02009B1H02.f1	RPS6KA5	Ribosomal protein S6 kinase, 90 kDa, polypeptide 5				−1.4		
SB03032B1E02.f1	SMARCAL1	SWI/SNF, matrix associated, regulator of chromatin			−5.6			
**SB03034B2D05.f1**	**ZBTB16**	**Zinc finger and BTB domain containing 16** *		**−1.6**	**1.9 (2.2)**		**1.9**	**1**
SB03036B1C02.f1	ZBTB16	Zinc finger and BTB domain containing 16*		−1.4	1.7		1.7	1.1
**3. Cell attachment and filopodial extension**								
SB02030A2H08.f1	CDH13	Cadherin-13 precursor	−1.4					
SB03003A1F12.f1	ITGA6	Integrin, alpha 6	1.4					
SB02022A2D12.f1	FNBP1L	Formin binding protein 1-like			1.2	−1.2	1.2	−1.2

Representative cDNA spots significant with cell-means model (p<0.01). Significant fold changes are shown under each pairwise comparison. See tables S1-5 for complete listing. Those in bold were chosen for qPCR validation and significant results (p<0.05) are shown within parenthesis.

* ZBTB16 primers were designed outside above EST clone sequences but within sequence annotated as ZBTB16 in zebra finch genome. SC: spring control; AC: autumn control; SE: spring STI; AE: autumn STI; Int: interaction effect (spring: SE vs. SC, autumn: AE vs. AC).

### Validation of Microarray Results by Real-Time RT-PCR

Nine genes of functional interest were selected for validation by quantitative PCR (qPCR) analysis (Table 2). Eight genes showed differential expression at p<0.01 in the microarray and were of interest in potential relationship to regulation of GnRH: glycoprotein hormone alpha chain precursor (*CGA*), transthyretin (*TTR*), mu-crystallin (*CRYM*), heat shock 70 kDa protein (*HSPA2*), zinc finger and BTB domain containing 16 (*ZBTB16*), and growth arrest and DNA damage-inducible, beta (*GADD45B*), vasoactive intestinal peptide (*VIP*), and neurensin 1 (*NRSN1*). In addition, c-fos (*FOS*) was also investigated since previous study in our laboratory showed differential expression upon territorial intrusion between two different seasons, although it was not significantly different in the array analysis.

**Table 2:** Primer sequences used for real-time PCR validation.

Target gene	Accession #	Forward primer	Reverse primer	Product size (bp)
*CGA*	CK310677.1	5′-CAGATCATGGATTGCTATGGG	5′-CCCTGCATGAGAAACTCTCC	107
*TTR*	CK302433.1	5′-CTGTTGATTCCAAATGCCC	5′-CATTAGCAGTGAACACCAC	282
*CRYM*	DV960559.1	5′-CCACATCAATGCTGTTGGAG	5′-AGAGCAGCATCTCTGGAATC	102
*HSPA2*	DV959345.1	5′-AAGGGCCAGATTCAGGAGAT	5′-TGTTGAGCTCTTTGCCATTG	100
*ZBTB16*	XM_002189445.1	5′-TGAAGACAGAGAGCAGAGCC	5′-TGAGCCAGTAAGTGCATTCG	178
*GADD45B*	CK304105.1	5′-ATCTGCACTGCATCCTCATC	5′-CAACAAAGGTTTGAGCCTCC	173
*VIP*	DV958568.1	5′-AGAATGCCATTTGATGGAGC	5′-AGAGTGGCGTTTGACAGGAC	162
*NRSN1*	CK303279.1	5′-GCACAGCTTCAATTTGGGAG	5′-TAAAAGCCGTCCCAGAGATG	108
*FOS*	CK304505.1	5′-AGGACTTCTGCACCGACCT	5′-GGGCCACTGAAGAGATGAGA	120
*ACTB*	CK307381.1	5′-CACAGCTGCCTCTAGCTCCT	5′-CAGGACTCCATACCCAGGAA	132

Glycoprotein hormone alpha chain precursor (*CGA*), transthyretin precursor (*TTR*), μ-crystallin (*CRYM*), heat shock 70 KDa protein (*HSPA2*), zinc finger and BTB domain containing 16 (*ZBTB16*), growth arrest and DNA-damage-inducible beta (*GADD45B*), vasoactive intestinal peptide (*VIP*), neurensin 1 (*NRSN1*), c-fos (*FOS*), and β-actin (*ACTB*). *ACTB* was used as internal control.

**Figure 5:**
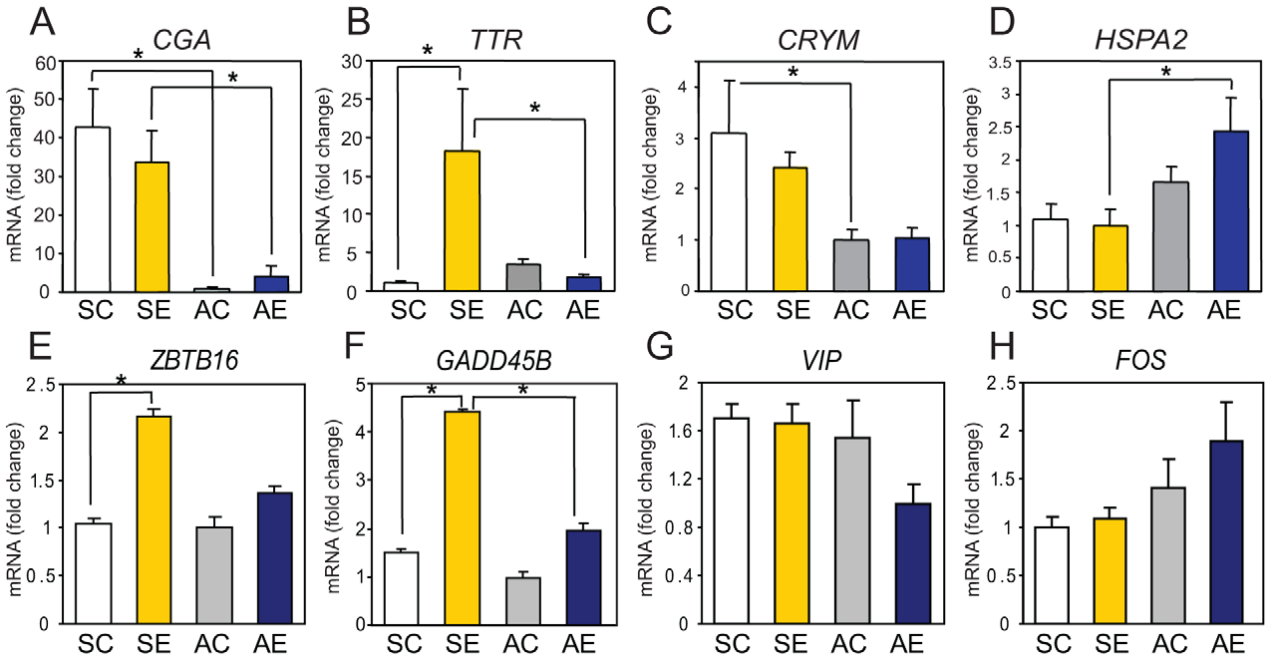
Validation of microarray results by real-time PCR. Expression of A) glycoprotein hormone alpha chain precursor (*CGA*), B) transthyretin (*TTR*), C) *μ*-Crystallin (*CRYM*), D) heat shock 70 KDa protein (*HSPA2*), E) zinc finger and BTB domain containing 16 *(ZBTB16*), F) growth arrest and DNA-damage-inducible, beta (*GADD45B*), G) vasoactive intestinal peptide (*VIP*), and H) c-fos (*FOS*) were determined. Results were calculated in relative RNA amounts using β-actin (*ACTB*) as internal control and shown as mean fold-change to group with lowest expression value. n = 5−7/group, * p<0.05 by ANOVA followed by Tukey's test as posthoc pair-wise comparisons. Results are shown in mean±SEM.

*CGA*, which showed large decreases in the autumn samples compared to the spring samples by microarray, also showed similar effects by qPCR (Table 1; 1-way ANOVA, F_(3,24)_ = 30.76, p<0.01). The qPCR assay confirmed an especially large decrease (42.8-fold) in AC vs. SC (Fig. 5A, Tukey's, p<0.01) and also a significant decrease (8.3-fold; Tukey's, p<0.01) in AE vs. SE.

*TTR* showed a complex pattern of regulation by microarray, up-regulated by STI in spring but down-regulated by STI in autumn (Table 1) and with a large seasonal differences in the control conditions (AC>SC). With qPCR, the difference in AC vs. SC was not significant, although there was a trend towards an increase consistent with the microarray data. However, qPCR did confirm a significant interaction effect of seasonal and social factors (2-way ANOVA, F_(1,20)_ = 7.62, p<0.05), with an increase by 18.1-fold in SE vs. SC and a decrease by 11.0-fold in AE vs. SE (Fig. 5B, Tukey's, p<0.05).

In the array, *CRYM* showed significant seasonal decreases in both AC vs. SC and AE vs. SE (Table 1). This seasonal effect was also significant by qPCR (1-way ANOVA, F_(3,24)_ = 5.98, p<0.01) with 3.1-fold decrease in AC vs. SC (Fig. 5C, Tukey's, p<0.05). Conversely, *HSPA2* was increased in both AC vs. SC and AE vs. SE by the microarray (Table 1). A significant seasonal increase was confirmed for *HSPA2* (ANOVA, F_(3,24)_ = 4.25, p<0.05) in AE vs. SE (2.4-fold, Tukey's, p<0.05) by qPCR, although the increase was not significant in AC vs. SC (Fig. 5D).

*ZBTB16* showed a seasonal/social interaction effect in the array data (up-regulated by STI in both seasons but greater response in spring; Table 1). The qPCR results (1-way ANOVA, F_(3,24)_ = 6.13, p<0.01) validated the up-regulation response by STI in spring (2.2-fold; Fig. 5E; Tukey's, p<0.01) and also showed a trend towards a decrease in AE vs. SE (although not significant). The statistical interaction effect fell short of significance by qPCR (2-way ANOVA, F_(1,24)_ = 3.07, p = 0.093). *GADD45B* also showed a seasonal/social interaction effect in the array data (up-regulated by STI in both seasons but response is greater in spring; Table 1). The qPCR results validated this (1-way ANOVA, F_(3,24)_ = 16.82, p<0.01) and showed a 3.0-fold increase in SE vs. SC, a 2.3-fold decrease in AE vs. SE (Fig. 5F), and a significant interaction effect (2-way ANOVA, F_(1,24)_ = 6.67, p<0.05). For two other genes, *VIP* and *NRSN1*, qPCR failed to confirm the effects observed by the microarray (Fig. 5G and data not shown for *NRSN1*).

Unexpectedly, expression of *FOS* mRNA was not significantly affected in the microarray data, although our previous observation using immunohistochemistry showed an increase of protein expression in hypothalamic nuclei by STI. To verify these microarray results, qPCR was performed in addition to the other selected 8 genes. However, *FOS* was not significantly affected either by season or STI (Fig. 5H), consistent with the lack of effect in the array analysis.

In summary, in 7 of 9 cases, qPCR generally confirmed most of the effects observed by microarray. *TTR*, *ZBTB16*, and *GADD45B* all showed significant or trends of interaction effect between season and social stimulus, whereas *CGA*, *CRYM*, and *HSPA2* showed consistent seasonal effects or trends under both social conditions.

## Discussion

Song sparrows display similar behavioral responses to STIs in breeding and non-breeding seasons. However, while there is social activation of the hypothalamo-pituitary-gonad axis resulting in an increase on testosterone secretion during breeding season, there is no such response to STIs in the non-breeding season despite very similar behavioral responses. Mechanisms of how increases in LH and T occur during territorial aggression exclusively in the breeding season have not been deciphered to date. Therefore, this study was designed to decipher potential molecular mechanisms underlying neuroendocrine responses during territorial aggression by global gene expression analysis. Our studies detected a large number of cDNAs (727) that are differentially expressed in the hypothalamus according to season or STI. For the most part, these 727 cDNAs represent non-redundant genes, as 555 have now been mapped to defined gene models in the zebra finch genome assembly (Ensembl Release 53, http://www.ensembl.org) and 513 (92% of the mapped genes) of which are unique.

Previous unpublished findings from our laboratory showed that the protein product of FOS is increased by STI in the spring but not in the autumn in hypothalamic regions important for neuroendocrine regulation. Because of this finding, our original hypothesis was that exposure to an STI will activate more genes in the hypothalamus in the breeding (spring) than in the non-breeding season (autumn). However, we found just the opposite - fewer cDNAs were differentially expressed upon territorial intrusion in spring than in the autumn. At a significance threshold of p<0.01, we only detected 67 cDNA spots that were affected by STI in the spring, but 173 cDNA spots were responsive to STI in the autumn. In addition, there were even larger differences in cDNA expression between the spring and the autumn birds independent of territorial challenge.

The large seasonal difference in number of mRNA transcripts affected by social interactions suggests that the system may be optimized in spring for a rapid neuroendocrine response requiring fewer gene expression changes, and that season-dependent basal gene/protein expression levels are likely to play a major role in how the hypothalamus responds to territorial intrusion. We speculate that photoperiod signals may act on the hypothalamus to establish this refined pathway, whereas socially-mediated increases in gonadally derived testosterone may feed back on the hypothalamus and other brain regions to assist in maintaining it through the spring.

Moreover, gene responses to territorial challenge in the autumn may represent functional activation of the hypothalamus relating to local production of T and estradiol, which are suggested to play an important role in displaying aggression during the non-breeding season [18], [19], and/or may represent suppression of neuroendocrine responses, although there was no direct evidence of this found from the array analysis. The different seasonal responses to STI could also be linked to parameters associated with a change of *plasticity* in the system between the two seasons. The LH/T signaling pathway is relatively quiescent in the non-breeding period. However, at this time it may be especially sensitive (and show increased *plasticity*) to environmental cues that will eventually lead to the seasonal differentiation in anticipation of the ensuing breeding season.

Although our overall conclusions are robust, there are several caveats to consider. We used microarray platform based on a different songbird species (zebra finch) to measure gene expression in the song sparrow. Comparative genomic DNA hybridization analysis have confirmed that most of the cDNAs on this array are equally effective for probing either zebra finch or song sparrow sequences, although a small percentage of probes hybridized poorly with song sparrow compared to zebra finch [21]. Removing these probes from our analysis (i.e. those showing a 2-fold decrease in signal with song sparrow compared to zebra finch DNA) did not significantly affect the outcome, supporting the reliability of the microarray result. We also note that some genes are represented on this array by multiple non-overlapping cDNA clones [21] (and see Tables S1, S2, S3, S4, and S5). However, we generally observed good concordance in the behavior of different cDNA clones derived from the same gene, and we do not think this significantly affects our overall conclusions. Importantly we were able to confirm the microarray data for 7 of 9 probes using qPCR – a validation rate similar to that observed in other experiments using SoNG Initiative microarray analysis pipeline [22], [23], [24], [25].

Another limitation to the present study is that whole hypothalamus was used instead of specific regions. This could result in dilution or masking of highly localized patterns of gene regulation within sub-regions of the hypothalamus. This may account for the lack of effect on *FOS* in our studies here, which contrasts with our previous results where an effect was detected using immunohistochemistry to examine tissue sections via microscopy. Alternatively, FOS expression may only be post-trascriptionally regulated. This underscores importance of further studies using *in situ* hybridization for more precise mRNA localization in tissues that are as complex as hypothalamus.

However, since not all gene-results were validated by qPCR, results from this microarray experiment should be interpreted with care. In our interpretations, we focused on genes that showed consistent trends with different cDNA spots and were discovered by functional pathway analysis. Thus the discussion will now focus on genes that showed significant differences, including those that regulate thyroid hormone action and neuroplasticity, which may, in turn, regulate GnRH secretion.

### Regulation of Thyroid Hormone

In this study, microarray expression analysis in conjunction with functional pathway analysis and qPCR showed that genes related to the regulation of thyroid hormones were significantly affected in both seasonal and STI comparisons. This is of considerable interest for several reasons. Most seasonal breeders at mid- to high-latitudes use changes in day length (photoperiod) as a cue to transition from one life history stage to another, such as from non-breeding to breeding stages. Thyroid hormones are known to play an important role in this process [26], [27], [28]. Exogenous thyroid hormone treatment has been shown to mimic effects of a long-day, inducing gonadotropin-releasing hormone (GnRH) secretion by affecting neuro-glial plasticity at the median eminence (ME) [29]. On the other hand, thyroidectomy inhibits termination of breeding and prevents gonadal regression after the development of photorefractorines in birds and rams [30], [31]. Down-regulation of thyroxin binding proteins such as transthyretin (TTR), T_4_-binding globulin, and albumin, has been associated with reduced hypothalamic T_4_ uptake and reproductive photorefractoriness in Siberian hamsters (*Phodopus sungorus*) [32]. More recently, it has been shown that thyroid-stimulating hormone (TSH) expressed in the *pars tuberalis* (PT) of the pituitary gland, induces type 2 deiodinase (DIO2), an enzyme that is critical for conversion of T_4_ into an active T_3_ form, in the hypothalamus of Japanese quails, *Coturnix japonica* [26].

**Figure 6:**
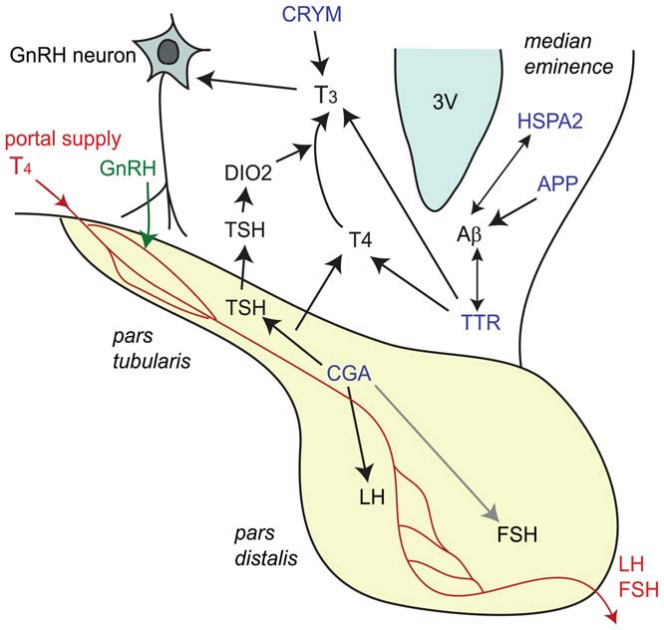
Schematic diagram showing the regulation of thyroid hormone action in *pars tuberalis* of pituitary and hypothalamus. Blue indicates genes that were significantly affected in the current microarray study: common glycoprotein alpha subunit (CGA), transthyretin (TTR), μ-crystallin (CRYM), and heat shock 70 KDa protein (HSPA2), and amyloid-β precursor protein (APP). TSH: thyroid stimulating hormone, DIO2: type-2 deiodinase, LH: luteinizing hormone, FSH: follicular-stimulating hormone, 3 V: third ventricle, T_3_: L-triiodothyronine, T_4_: thyroxine.

In our analysis, we observed a large and significant difference in the basal expression of the *CGA* mRNA in autumn vs. spring. CGA protein forms heterodimers with hormone-specific β-subunits, including luteinizing hormone β (LHβ), follicle-stimulating hormone β (FSHβ), and TSHβ. Lack of α-subunit will lead to an inhibition of heterodimer formation and severe hypogonadism and hypothyroidism will result [33]. Since it has been reported previously that *CGA* expression is restricted to the PT of the pituitary in adult chickens [34] and is not seen in other areas of the hypothalamus, this expression difference probably occurs at the level of the PT, which was consistently included in all hypothalamic samples in this study. TSHβ expression is located in the PT of rats, hamsters, and other mammals [35], [36], [37]. Therefore, increases of *CGA* expression in spring vs. autumn may increase the synthesis of TSH, and subsequently influence *in situ* production of T_3_ by affecting DIO2 in the ME [26] (Fig. 6). T_3_ affects GnRH secretion [29] leading to LH release by the *pars distalis* (PD) of the anterior pituitary (Fig. 6). However, *DIO2* was not significantly affected in this microarray. Thus, further investigation needs to be done using *in situ* techniques to verify the expression of *DIO2* or consider alternative ways by which an increase of CGA may lead to elevated release of LH and T. On this note, LHβ has also been found to be expressed in PT of sheep [38]. Therefore, it may also be possible that LH expression is directly affected by increased CGA expression (Fig. 6). The increase of LH upon STI in the breeding season may be largely due to this difference in basal expression of *CGA*. VIP, which is known induce the transcription of CGA in αT3-1 cells, was also downregulated in AC vs. SC in the array, although this could not be confirmed with qPCR.

Two other genes related to thyroid hormone function were significantly affected by season in this study; *TTR* and *CRYM*. Although it was not confirmed by qPCR statistically, *TTR* was increased in AC vs. SC. *CRYM* was decreased in AC vs. SC. TTR is a transmembrane transporter for thyroid hormone as well as for retinols. Down-regulation of this gene during reproductive refractoriness to short days in Siberian hamster was associated with reduced hypothalamic T_4_ uptake [32]. Increases of this gene in autumn in the song sparrow may therefore suggest increased T_4_ uptake at the hypothalamus. TTR is also known to interact with T_3_ in bullfrogs during metamorphosis [39] and is involved in the efflux of T_3_ [40] (Figs. 4A and 6). However, the physiological significance of this is yet unknown, and seems somewhat contradictory since basal expression of LH is decreased in autumn. *TTR* mRNA expression was also increased in white-crowned sparrow (*Zonotrichia leucophrys gambelii*) telencephalon in the non-migratory condition vs. migratory condition [41], but the physiological relevance of this regulation is also yet unknown.

*TTR* had interaction effect in the microarray showing up-regulation by STI in spring and down-regulation by STI in autumn. The qPCR confirmed this trend and there was a clear increase of *TTR* expression by STI in spring by qPCR. In this case, an increase of *TTR* leading to increased hypothalamic T_4_ uptake may at least partially explain the subsequent surge of LH and T following aggressive interactions. It may be that social interaction affects thyroid hormone action in a similar way as changes in photoperiod do.

*CRYM* is also known as reduced nicotinamide adenine dinucleotide phosphate (NADPH)-dependent 3,5,3′-triiodo-L-thyronine (T_3_)-binding protein, and is expressed in retina, brain, heart and kidney [42]. It binds T_3_ and thereby increases the cellular uptake and decreases the cellular efflux rate of T_3_ and suggesting a role of this protein to retain T_3_ in the intracellular cytoplasm [43]. Higher concentration of this protein in the spring is consistent with critical activation of thyroid receptor during the breeding season. Moreover, high mobility group nucleosomal binding domain 3 (HMGN3), which binds to thyroid hormone receptor beta in the presence of T_3_ [44], had a significant interaction effect (upregulated in spring and downregulated in autumn with STI; Table 1). This family of proteins helps alter chromatin structure and enhances transcription [45] and may also have functional relationship to regulation of GnRH release.

HSPA2 belongs to the large group of the heat shock protein 70 family, members of which generally function as molecular chaperones to stabilize the folded state of proteins, and control protein disaggregation [46]. *HSPA2* was increased in AC vs. SC in microarray and a similar trend was observed with qPCR although it was not significant. Up-regulation of this cDNA spot in AE vs. SE with microarray was confirmed with qPCR. It has been reported that heat shock proteins increase phagocytosis of amyloid-β protein by microglial activation [47]. Therefore differences in *HSPA2* may be regulating Aβ levels in the autumn season. Amyloid-β precursor protein (APP) is a cell surface receptor and trans-membrane precursor protein that can be cleaved by secretases to form various types of peptides. The β secretase releases amyloid-β protein that is associated with the plaques observed in Alzheimer's disease [48].

It has also been shown that *TTR* interacts with and potentially sequesters amyloid-β protein to prevent amyloid formation in cerebrospinal fluid [49] and in kidney [50]. Mice lacking *TTR* have memory deficits and this was reversed by retinoic acid treatment [51]. Moreover, mice with over-expression of *APP* and mutation of presenilin (a risk factor for familial Alzheimer's disease) have increased production of amyloid-β protein and display enhanced territorial aggression when compared to wildtypes [52]. There was a slight but significant increase of *APP* in AE vs. AC in the array. Due to these potential and interesting relationships of APP, Aβ and aggression, it may be of interest to investigate the concentration of amyloid-β protein with/without STI in hypothamali of these birds.

### Neuroplasticity and Transcriptional Regulations

Some changes in expression that we detected suggest an increase in neuroplasticity in the autumn vs. spring, and upon STI in autumn. There were increases in expression of the ribosomal protein-coding gene (*MRPL12*) and ribosomal protein S6 kinase (*RPS6KA2*) in AC vs. SC. Expressions of mRNA for two ribosomal proteins (RPL3 and MRPS17) were increased in AE vs. AC. Expressions of two ribosomal protein kinases (*RPS6KA2* and *RPS6KA5*) were decreased in AE vs. AC. PAP-associated domain containing 1 (*PAPD1*), also known as mitochondrial poly(A) polymerase (MTPAP) is known to play a role in mitochondrial RNA processing [52] and was increased in AE vs. AC (Table 1 under *Neuroplasticity and transcriptional regulation*).

Ribosomal proteins have critical function in ribosome biogenesis and activity and several ribosomal proteins have been shown to have extra-ribosomal functions in apoptosis, DNA repair, transcription and translation in mammalian cells (reviewed in [53]). Ribosomal protein S6 kinase controls various cellular processes such as cell proliferation, cell death, and cell growth [54] and may be required for synaptic plasticity important for cognitive processing [55]. Major changes in gene expression for ribosomal and mitochondrial proteins were also observed in a recent study of song response habituation in the zebra finch, a model of acute memory formation [22], [56]. Thus, changes in the expression of mRNA coding ribosomal proteins and ribosomal protein kinases may relate to increase in neuroplasticity in the autumn in general and upon STI. This may have resulted in the increased number of genes significantly different with STI in the autumn vs. spring. When spring comes, the system may have already reached a stable activated state sufficient to support rapid neuroendocrine signaling, which is less dependent on acute changes in gene expression.

Another group of genes suggested an increase in neuroplasticity in autumn. Light intermediate chain of the axonemal dynein (DNALI1) and light chain of the cytoplasmic dynein (DYNLL1) was upregulated in AC vs. SC in the array. Dyneins are microtubule-based motor proteins and are divided into two groups; axonemal and cytoplasmic dyneins. Axonemal dyneins are important in the sliding of microtubules in the axonemes of cilia and flagella-like structures and found in cell types that have these structures [57]. Ependymal cells of the third ventricle in the basal hypothalamus contain apical cilia and/or microvillous processes [58]. Cytoplasmic dyneins are important in various activities including intracellular transport and nuclear migration [57]. The increased expression of these motor proteins perhaps suggest a reciliation, cell renewal, and/or increase in transport activities of cellular components in these ependymal and other cell types in autumn.

Expression of cDNA coding *NRSN1* which plays an essential role in neurite extension during nervous development, regeneration, and plasticity [59], was up-regulated in AC vs. SC in the microarray. This also fits well with a potential of increased neuroplasticity in autumn compared to spring, although this increase was not validated with qPCR, for a reason unknown. Transcripts of *GADD45B* are usually increased during stressful growth arrest conditions and treatment with DNA-damaging agents (reviewed in [60]). Differential expression of this gene had a seasonal/social interaction effect which suggested an increase with STI in both seasons but with a stronger effect in the spring. Increase in activity of GADD45B is known to increase DNA demethylation of genes that are necessary for neurogenesis [61], [62], [63]. This may suggest that repeated territorial intrusion may affect neural plasticity in both seasons, but more effectively in the spring for certain types of plasticity that this gene regulates, in contrast to perhaps a more general plasticity increased by ribosomal related genes in AC vs. SC and AE vs. AC.

There were other genes that regulate transcription that were affected in the study. In addition to *HMGN3* (discussed in the previous “thyroid hormone” section), which helps to alter chromatin structure and enhances transcription [45], SWI/SNF related, matrix associated, actin dependent regulator of chromatin, subfamily a-like 1 (SMARCAL1), which also regulates the transcription of certain genes by altering chromatin structure [64], and was down-regulated by 5.7-fold in spring with STI. *ZBTB16* is involved in cell cycle progression, interacts with a histone deacetylase, and act as repressor of gene transcription [65]. This gene was increased by STI in spring. DNA methyltransferase 1 associated protein 1 (DMAP1) that is known to bind to DNA methyltransferase (DNMT1) and be involved in transcriptional repression [66] was also differentially regulated by interaction (up in spring with STI and down in autumn with STI) in this study. These facts fit well with the fact that more genes were altered in autumn with STI compared to spring, but also suggest that this transcriptional regulation may be of epigenetic origin.

### Cell Attachment and Filopodial Extension

It has become evident that GnRH secretion is regulated at least partly by non-neuronal cells including tanycytes (type of ependymal cells), astrocytes and endothelial cells [67]. Tanycyte endfeet completely encase the basal lamina of the median eminence and show tight cell-to-cell interaction to prevent the GnRH nerve terminals from reaching the capillaries of the hypothalamo-pituitary blood portal system under short days (SD). This encasement recedes in long day (LD) in Japanese quail (*Coturnix japonica*) [68] and after thyroid hormone implants [29], thereby potentially allowing GnRH secretion.

There were several genes affected in the array that could be involved in cell-cell interactions and morphological changes relating to GnRH neurons and their surrounding cells. Integrin alpha 6 (ITGA6) was upregulated in autumn compared to spring. Integrins are cell-surface receptors that mediate the attachment and cell signaling of a cell to other cells or to the extracellular matrix. The increase of integrin in autumn may be consistent with this close interaction of the tanycyte endfeet. On the other hand, cadherin-13 (CDH13) is another cell-cell adhesion glycoprotein and was down-regulated in AC vs. SC. CDH13 has been suggested to function as a negative regulator of neural cell growth [69]. Ependymal cells do not express CDH13 protein [69]. Therefore, decrease of this cadherin in autumn may suggest weaker cell-cell adhesion in other cell types (e.g. neurons, astrocytes) to allow neural plasticity to prepare for the spring.

During the GnRH/LH surges, GnRH nerve endings are allowed to directly contact the pericapillary space by filopodial extension of nerve terminal or by evaginations of basal lamina to the neural parenchyma in the median eminence [68], [70]. Formin binding protein like-1 (FNBP1L, also known as Toca-1) which has been shown to induce filopodia and neurite formation [71], was upregulated with STI in spring but downregulated by STI in fall and was the only gene that also showed a significant interaction. These findings suggest that LH increase upon STI in the spring may also be regulated by morphological changes of tanycytes as well as filopodial extensions of GnRH neurons. Additional in situ studies will clarify where in the hypothalamus these genes are being regulated.

### Conclusions

Among all pairwise comparisons, the number of cDNAs significantly affected was highest between the two control groups in autumn and spring. It is likely that changes in basal expression between seasons are major contributors to the difference in neuroendocrine response upon aggression. Of the cDNAs differentially expressed, those involved in the regulation and activity of thyroid hormone, neuroplasticity, transcription, cell-adhesion and neurite extension were of special interest due to their potential relationship with the regulation of GnRH. However, determining the *in situ* expression of these differentially-regulated-genes in the specific regions of hypothalamus, in addition to expressions of related genes/proteins that were not in the spotted array, may help further clarify some of the mechanisms underlying seasonal differences in the neuroendocrine response to territorial challenge in this species.

## Methods

### Animals

Free-ranging male song sparrow (*Melospiza melodia morphna*) were captured in the following 7 sites in western Washington State (48°N): Crescent Lake, Cherry Valley, Prison Farm, Spencer Island (all in Snohomish county), Skagit Valley (Skagit county), Pack Forest (Pierce county), Stillwater (King county). Birds were captured during 1) the breeding season (mid to late June, 2005; referred to as “spring” in this paper) and 2) the non-breeding season (mid November, 2005 to late January, 2006; referred to as “autumn” in this paper) (n = 7−8). All animal procedures were approved by the Institutional Animal Care and Use Committee of the University of Washington and were conducted in accordance with the NIH *Guide for the Principles of Animal Care*. Permits to capture free-ranging birds using mist nets were obtained from U.S. Fish and Wildlife Service and Washington State Department of Fish and Game.

### Simulated Territorial Intrusion (STI) and Behavioral Data

A live caged male song sparrow decoy was presented with recorded male conspecific songs to a free-ranging territorial male. For controls, a live caged male white-crowned sparrow decoy with white-crowned sparrow songs was used. During the first 10 minutes of the STI, the following behaviors were recorded from the focal bird: 1) number of songs, 2) number of flights directed at the decoy, 3) closest approach to the decoy, and 4) time spent within 5 meters (m) of the decoy. The STI was continued for additional 20 min and then birds were quickly captured with a mist net.

### Tissue Collection

Immediately after capture, each bird was anesthetized with isoflurane (10%) and decapitated. Hypothalamus was quickly dissected out and quickly frozen on dry ice and subsequently stored at −80°C. Due to the location and structural association of the *pars tuberalis* in avian species, it was impossible to accurately dissect out *pars tuberalis* from the hypothalamus, therefore all samples were collected along with *pars tuberalis* for consistency; however, other parts of the pituitary were removed.

### Microarray Hybridization

Methods of sample preparation and microarray hybridization have been described previously [21]. Briefly, total RNA was isolated using TRI Reagent (Ambion, Austin, TX), treated with TURBO DNase (Ambion) and purified with RNeasy Mini spin columns (Qiagen, Valencia, CA). Then 0.5 µg of DNase treated total RNA was used for oligo (dT) primed reverse transcription followed by cRNA amplification step using Low RNA Input Fluorescent Linear Amplification Kit (Agilent, Santa Clara, CA) and 1 µg of the resulting amplified RNA was used in the subsequent RT reactions using an indirect aminoallyl incorporation protocol with Cy3/Cy5 dye labeling (GE Life Sciences, Piscataway, NJ). Labeled cDNA was hybridized overnight at 42°C to the custom-made zebra finch brain spotted cDNA microarrays, using the resources of Songbird Neurogenomics (SoNG) Initiative, as part of Community Collaborations #25 [21]. Each experimental song sparrow sample (total of 31, n = 7−8/group) was hybridized along with a universal reference sample consisting of brain cDNA from 30 non-breeding zebra finches [21] using a dye-swap reference design [72]. Then the slides were washed and scanned using Axon GenePix 400B (Molecular Devices, Sunnyvale, CA) and images were visualized by GenePix Pro 6.0 (Molecular Devices). Images were screened to identify aberrant spots (e.g. dust spots, scratches), which were then flagged to be excluded from further analysis.

### Microarray Statistics and Pathway Analysis

Data pre-processing and statistical analysis were done in R using the limma package [73] from Bioconductor (www.bioconductor.org). Spot intensity values were calculated by subtracting the local median background from the median foreground value, and adjusting negative/zero values to 0.5 [74]. Normalization was carried out in two steps: first a print-tip loess within-array normalization to remove dye biases, then a between-array scale normalization to remove array biases [75]. However, even after these standard normalization steps, hierarchical clustering indicated a persistent batch effect due to array print batch combined with the channel in which the universal reference sample was. This batch effect was accounted for in the statistical model using the average correlation within the blocks [76]. Because pairwise-comparisons between treatment groups (AC vs. SC, AE vs. SE, SE vs. SC, and AE vs. AC) and the interaction effect were of interest, not the main effects of season or treatment, a cell-means model (Y_1_ = a_1_x_i1_+a_2_x_i2_+a_3_x_i3_+a_4_x_i4_+e) was used instead of a 2-way ANOVA. However, the results for the pairwise comparisons and interaction from the cell-means model are identical to those calculated from a 2-way ANOVA [77]. Limma also applies an empirical Bayes correction to moderate the standard errors based on information from all cDNAs; these moderated t-statistics also have increased degrees of freedom, representing the extra information gained by borrowing information across cDNAs [77]. Multiple testing correction methods were not applied since they depend heavily on the assumption that each cDNA expression is independent and can often be too restrictive as a result of their effort to prevent all type-I errors, thus filtering out truly regulated spots [78]. Instead, a threshold of raw p-value <0.01 was used for significance and a subset of genes was validated by real-time qPCR. Microarray data generated in this study comply with the Minimum Information about Microarray Experiment (MIAME) guidelines and original gpr files are available at Gene Expression Omnibus (accession#GSE18970). Significantly affected cDNA spots were clustered hierarchically by Pearson correlation using GeneSpring GX (Agilent, Santa Clara, CA). They were also imported to IPA software (Ingenuity Systems, Redwood City, CA) for functional gene network analysis.

### Real-Time Quantitative PCR

Groups of genes of interest, e.g. genes that regulate thyroid hormone, neuroplasticity, and gene transcription were selected to perform real-time PCR to validate microarray results. The total RNA samples (also used for the microarray hybridizations) were quantified using NanoDrop ND-1000 (NanoDrop Technologies, Wilmington, DE) and 500 ng was reverse transcribed for 50 min at 42°C in a 20-µl reaction with 200 U of SuperScript II reverse transcriptase (Invitrogen, Carlsbad, CA) and 0.5 µg of Oligo (dT)_12–18_ primer following the manufacturer's protocol. The synthesized cDNA was diluted 1∶25, and 2 µl of this diluted cDNA was used for subsequent 10 µl quantitative PCR (qPCR) reactions with 2x SYBR Green PCR Master Mix (Applied Biosystems, Foster City, CA) and 300 nM of each forward and reverse primer. Primers (Table 2) were designed with known zebra finch (*Taeniopygia guttata*) sequences submitted on GenBank and by using Vector NTI (Invitrogen, Carlsbad, CA). All primers except for *HSPA2* were designed across 2 exons to inhibit potential amplification of genomic DNA. Sequence of the cDNA clone that is annotated as *HSPA2* did not span an intron; therefore, intron-spanning primers could not be designed for this gene. However, appropriate negative controls were run with all qPCR runs to make sure that the product is not of genomic DNA origin. All primers except for ZBTB16 were designed using one of the EST sequences of the cDNA clone that was significant in the array. Both of the sequences for differentially expressed cDNA spots (SB03034B2D05.f1 and SB03036B1C02.f1) that are annotated for ZBTB16, actually blasted to a region that is outside coding region. Therefore, another sequence obtained from NCBI (XM_002189445.1) that does blast within the coding region of predicted zebra finch ZBTB16 was used.

Quantitative real-time PCR analysis was done using ABI Prism 7700 Sequence Detector (Applied Biosystems, Foster City, CA). The C_t_ (threshold cycle) value was obtained, and relative amount of amplicon was calculated using the relative standard curve method described in Applied Biosystems User Bulletin 2. In addition, some of the resulting PCR products were run on a 1.5% agarose gel, appropriate sized bands were excised, extracted with QIAquick Gel Extraction Kit (Qiagen, Valencia, CA), and sequenced to verify the sequence specificity for song sparrow. These sequences (>98% homologous to *T. guttata*) are available upon request.

### Statistics

Statistical analysis for real-time PCR was performed by 1-way ANOVA followed by post-hoc Tukey's test for pair-wise comparisons using SYSTAT 11 (SSI, Richmond, CA). Interaction effect was obtained separately by 2-way ANOVA. The data were transformed into natural log to meet the normality assumption for some data. Statistical analysis for behavioral data was performed by Kruskal-Wallis 1-way ANOVA followed by Mann-Whitney U test for pair-wise comparisons using the same program since normality assumptions were still violated after transformations of the data. All *p* values were two sided and considered statistically significant at *p*<0.05. Data are shown as mean±SEM.

## Supporting Information

Table S1 Complete list of cDNAs affected by the comparison AC vs. SC with cell-means model, p<0.01. The expressions in autumn control (AC) compared to spring control (SC) are shown in fold changes.
(0.02 MB PDF)

Table S2 Complete list of cDNAs affected by the comparison AE vs. SE with cell-means model, p<0.01. The expressions in autumn STI (AE) compared to spring STI (SE) are shown in fold changes.
(0.02 MB PDF)

Table S3 Complete list of cDNAs affected by the comparison SE vs. SC with cell-means model, p<0.01. The expressions in spring STI (SE) compared to spring control (SC) are shown in fold changes.
(0.01 MB PDF)

Table S4 Complete list of cDNAs affected by the comparison AE vs. AC with cell-means model, p<0.01. The expressions in autumn STI (AE) compared to autumn control (AC) are shown in fold changes.
(0.02 MB PDF)

Table S5 Complete list of cDNAs affected by the interaction (season×social) with cell-means model, p<0.01. The expressions in spring STI (SE) compared to spring control (SC) and autumn STI (AE) compared to autumn control (AC) are shown in fold changes.
(0.01 MB PDF)
