# Supplementary material for: Seasonal Differences of Gene Expression Profiles in Song Sparrow (Melospiza melodia) Hypothalamus in Relation to Territorial Aggression
Source: PLoS One. 2009 Dec 4;4(12):e8182. doi: 10.1371/journal.pone.0008182 (PMC2780717; doi:10.1371/journal.pone.0008182)
Supplement: Table S1 — Complete list of cDNAs affected by the comparison AC vs. SC with cell-means model, p<0.01. The expressions in autumn control (AC) compared to spring control (SC) are shown in fold changes. (0.02 MB PDF) [file pone.0008182.s001.pdf]

| Spot ID | UniGene ID | HGNC_symbol | Gene Description | Fold Change AC vs. SC | T Statistics | df | P-value |
|---|---|---|---|---|---|---|---|
| SB02006A2H05.f1 | Gga.28520 | CGA | Glycoprotein hormones alpha chain precursor | -15.96 | -10.16 | 29.26 | 0.0000 |
| SB03032A2G07.f1 | Gga.1079 | CRYM | Mu-crystallin homolog | -2.85 | -4.41 | 29.26 | 0.0001 |
| SB03046B1C04.f1 | Gga.1079 | CRYM | Mu-crystallin homolog | -2.73 | -4.81 | 29.26 | 0.0000 |
| SB03047B2E11.f1 | Gga.1079 | CRYM | Mu-crystallin homolog | -2.61 | -4.33 | 29.26 | 0.0002 |
| SB02007A1D12.f1 | Gga.2898 | SMTN | Smoothelin-like protein 1 | -1.83 | -5.98 | 29.26 | 0.0000 |
| SB02003B1B05.f1 | | | | -1.71 | -3.42 | 29.26 | 0.0019 |
| SB03021B2C08.f1 | | | | -1.57 | -3.56 | 29.26 | 0.0013 |
| SB03038A2F11.f1 | Gga.4000 | CHRNA7 | cholinergic receptor, nicotinic, alpha 7 | -1.54 | -4.24 | 29.26 | 0.0002 |
| SB02003A2D09.f2 | Gga.4605 | HNRPDL | Heterogeneous nuclear ribonucleoprotein A/B | -1.53 | -4.25 | 29.26 | 0.0002 |
| SB03043B1D06.f1 | Gga.33813 | VLLH2748 | Similar to VLLH2748 | -1.53 | -3.79 | 29.26 | 0.0007 |
| SB03042A1D05.f1 | Gga.666 | VIP | VIP peptides precursor | -1.47 | -4.33 | 29.26 | 0.0002 |
| SB010020000D02.B | Gga.14972 | LRRC3B | Leucine-rich repeat-containing protein 3B precursor | -1.47 | -2.91 | 29.26 | 0.0069 |
| SB03009B1E07.f1 | Gga.18849 | CD109 | CD109 molecule | -1.45 | -3.71 | 28.26 | 0.0009 |
| SB010007000A12 | Gga.23653 | UBR3 | biquitin protein ligase E3 component n-recognin 3 | -1.44 | -2.84 | 29.26 | 0.0080 |
| SB02040B1D06.f1 | Gga.9399 | YOD1 | HIV-induced protein-7-like protease, partial | -1.44 | -2.95 | 29.26 | 0.0062 |
| SB02031B2F04.f1 | | | | -1.43 | -3.97 | 29.26 | 0.0004 |
| SB03009B1A11.f1 | Gga.12655 | TRAF3 | TNF receptor-associated factor 3 | -1.40 | -3.26 | 29.26 | 0.0028 |
| SB03046A1G06.f1 | Gga.9559 | CTTNBP2NL | CTTNBP2 N-terminal like | -1.39 | -2.81 | 29.26 | 0.0087 |
| SB03025A1D09.f2 | Gga.4502 | SFRS5 | Splicing factor, arginine/serine-rich 5 | -1.39 | -2.97 | 29.26 | 0.0059 |
| SB02030A2H08.f1 | Gga.24273 | CDH13 | Cadherin-13 precursor | -1.38 | -2.95 | 29.26 | 0.0062 |
| SB02031A1B02.f1 | Gga.9946 | C22orf32 | Chromosome 22 open reading frame 32 | -1.38 | -2.91 | 29.26 | 0.0068 |
| SB010023000C07 | Gga.4845 | SFRS5 | Splicing factor, arginine/serine-rich 5 | -1.37 | -3.29 | 29.26 | 0.0026 |
| SB02013B1B11.f1 | Gga.12888 | HIPK2 | Homeodomain interacting protein kinase 2 | -1.36 | -3.01 | 29.26 | 0.0054 |
| SB03037B2G11.f1 | Gga.6172 | INPP5F | inositol polyphos-5-phosphatase F 1 | -1.35 | -4.43 | 29.26 | 0.0001 |
| SB03046A1H05.f1 | Gga.7811 | NME6 | Non-metastatic cells 6 | -1.35 | -3.76 | 29.26 | 0.0008 |
| SB03023B1C06.f1 | Gga.22158 | NACC2 | BTB (POZ) domain containing 14A | -1.34 | -3.25 | 29.26 | 0.0029 |
| SB02032A1G02.f2 | Gga.27049 | OPRL1 | TC199994 | -1.34 | -3.35 | 29.26 | 0.0022 |
| SB02038A2A01.f1 | Gga.11999 | SCG2 | Secretogranin-2 precursor | -1.34 | -3.15 | 29.26 | 0.0038 |
| SB03048B2H12.f1 | Gga.33929 | MAP4 | Microtubule-associated protein 4 | -1.33 | -3.28 | 29.26 | 0.0027 |
| SB03003A1E08.f1 | Gga.20687 | PLA2G10 | Group X secretory phospholipase A2 precursor | -1.33 | -2.83 | 28.26 | 0.0084 |
| SB03020A2H12.f1 | Hs.513695 | PDXDC2 | Pyridoxal-dependent decarboxylase domain containing 2 | -1.33 | -2.95 | 29.26 | 0.0062 |
| SB03005B1G10.f1 | | | | -1.32 | -3.57 | 29.26 | 0.0013 |
| SB03003A2B07.f1 | Gga.33929 | MAP4 | Microtubule-associated protein 4 | -1.32 | -3.17 | 29.26 | 0.0036 |
| SB03018B2F04.f1 | Gga.8855 | USP32 | Ubiquitin specific peptidase 32 | -1.32 | -2.81 | 29.26 | 0.0087 |
| SB010014000A06 | Gga.742 | SST | Somatostatin precursor | -1.31 | -3.80 | 29.26 | 0.0007 |
| SB02014A1G07.f2 | Gga.33766 | MAN2A2 | Alpha-mannosidase IIx | -1.31 | -2.86 | 29.26 | 0.0078 |
| SB02031B2D01.f1 | Hs.501857 | DENND5A | DENN/MADD domain containing 5A | -1.30 | -3.20 | 29.26 | 0.0033 |
| SB02008A1G03.f1 | Gga.15322 | MAPT | Microtubule-associated protein tau | -1.30 | -2.85 | 29.26 | 0.0080 |
| SB03040B2B03.f1 | Gga.33916 | GLRA2 | Glycine receptor alpha-2* chain precursor | -1.29 | -2.95 | 29.26 | 0.0062 |
| SB03021B1F10.f1 | Gga.10260 | SF4 | Splicing factor 4 | -1.28 | -3.10 | 29.26 | 0.0043 |
| SB03006B1A06.f1 | Gga.29178 | LACE1 | Lactation elevated 1 | -1.28 | -3.41 | 29.26 | 0.0019 |
| SB03025B1G06.f2 | | | | -1.28 | -2.82 | 29.26 | 0.0086 |
| SB03010B1A01.f1 | Gga.767 | MTNR1B | Melatonin receptor 1B | -1.28 | -2.84 | 29.26 | 0.0080 |
| SB010005001E09 | Gga.28965 | SPOP | Speckle-type POZ protein | -1.28 | -3.24 | 29.26 | 0.0030 |
| SB02044A2A04.f1 | | | | -1.27 | -3.16 | 29.26 | 0.0037 |
| SB03023A1G03.f1 | | | | -1.27 | -2.81 | 29.26 | 0.0088 |
| SB010021000E01 | Gga.10727 | PAPSS1 | Bifunctional 3'-phosphoadenosine 5'-phos | -1.27 | -3.17 | 29.26 | 0.0036 |
| SB02023A2E06.f1 | Gga.33945 | SETD8 | Glioma tumor suppressor candidate region | -1.27 | -2.89 | 29.26 | 0.0072 |
| SB03003A1E11.f1 | Gga.17826 | OXCT1 | 3oxoacid CoA transferase 1 | -1.27 | -4.20 | 29.26 | 0.0002 |
| SB03011B2D10.f1 | Gga.25774 | HS3ST5 | Heparan sulfate glucosamine 3-O-sulfotransferase 5 | -1.27 | -2.78 | 29.26 | 0.0095 |
| SB02041A2A05.f1 | Gga.4317 | CACNA1B | N-type calcium channel alpha-1B cdB1 variant | -1.27 | -3.00 | 26.26 | 0.0059 |
| SB03044B1H02.f1 | Gga.14699 | SH3D19 | SH3 domain protein D19 | -1.27 | -2.91 | 29.26 | 0.0069 |
| SB02044B2C12.f1 | Gga.23874 | RASGRF1 | RAS protein-specific guanine nucleotide-releasing factor 1 | -1.27 | -3.10 | 29.26 | 0.0043 |
| SB02048B2H01.f1 | Gga.5775 | HNRNPA3 | Heterogeneous nuclear ribonucleoprotein A3 | -1.27 | -4.25 | 29.26 | 0.0002 |
| SB03050B2D06.f1 | Hs.715029 | MFSD6 | Major facilitator superfamily domain containing 6 | -1.26 | -2.97 | 29.26 | 0.0058 |
| SB03045B2F12.f1 | Gga.616 | ADCYAP1 | Adenylate cyclase activating polypeptide 1 | -1.26 | -2.82 | 29.26 | 0.0085 |
| SB02026B2F01.f1 | Gga.13325 | FBXO9 | F-box only protein 9 | -1.26 | -3.08 | 29.26 | 0.0044 |
| SB02038B2E05.f1 | Gga.39962 | CLCC1 | Chloride channel CLIC-like protein 1 Precursor | -1.26 | -2.84 | 29.26 | 0.0082 |
| SB03047B2B10.f1 | Gga.42978 | MYCBPAP | MYCBP associated protein | -1.26 | -3.07 | 29.26 | 0.0046 |
| SB02040B2H08.f1 | Gga.11578 | GDI2 | Rab GDP dissociation inhibitor beta | -1.26 | -3.44 | 29.26 | 0.0018 |
| SB03018B1E05.f1 | Gga.43262 | POMT2 | Protein-O-mannosyltransferase 2 | -1.26 | -3.33 | 29.26 | 0.0023 |
| SB03026A2F12.f1 | Gga.39754 | RNMTL1 | Double C2-like domain containing protein | -1.26 | -2.87 | 29.26 | 0.0076 |
| SB03026B1G01.f1 | Gga.22590 | PGRMC1 | Progesterone receptor membrane component 1 | -1.25 | -2.79 | 29.26 | 0.0092 |
| SB02023B2H03.f1 | Gga.4000 | CHRNA7 | Cholinergic receptor, nicotinic, alpha 7 | -1.25 | -2.85 | 29.26 | 0.0079 |
| SB03045A2D08.f1 | | | | -1.25 | -3.02 | 29.26 | 0.0052 |
| SB02027B2E04.f1 | Gga.9984 | NBR1 | Next to BRCA1 gene 1 protein | -1.25 | -3.51 | 29.26 | 0.0015 |
| SB02035B2A03.f1 | Mm.93335 | OMG | Oligodendrocyte myelin glycoprotein | -1.25 | -3.65 | 29.26 | 0.0010 |
| SB03013B1B03.f1 | Gga.26211 | FAM89A | Dihydroxyacetone phosphate acyltransfera | -1.25 | -3.19 | 29.26 | 0.0034 |
| SB02023A2E11.f1 | Gga.42839 | INSR | Insulin receptor | -1.25 | -2.81 | 29.26 | 0.0088 |
| SB02027B1G10.f1 | Gga.6131 | KPNA3 | Karyopherin alpha 3 subunit | -1.25 | -3.09 | 29.26 | 0.0044 |
| SB02007A2F11.f1 | Gga.1785 | GPR177 | G protein-coupled receptor 177 | -1.25 | -2.80 | 29.26 | 0.0090 |
| SB02020B2H10.f1 | Hs.486361 | RLBP1L2 | Retinaldehyde binding protein 1-like 2 | -1.24 | -2.93 | 29.26 | 0.0066 |
| SB02007B2D06.f1.A | | | | -1.24 | -2.96 | 29.26 | 0.0060 |
| SB03044B1B07.f1 | Gga.21204 | HSPA4L | Heat shock 70kDa protein 4-like | -1.24 | -3.16 | 29.26 | 0.0037 |
| SB02046A2F04.f1 | | | | -1.24 | -2.79 | 29.26 | 0.0093 |
| SB03050A1H10.f1 | Gga.23309 | CACNB2 | Voltage-dependent L-type calcium channel | -1.24 | -3.08 | 29.26 | 0.0045 |
| SB02042A2G12.f1 | Gga.2749 | ZNF462 | Zinc finger protein 462 | -1.23 | -3.00 | 29.26 | 0.0055 |
| SB02013B2B10.f1 | Gga.4933 | HIF1A | Hypoxia-inducible factor 1, alpha subunit | -1.23 | -2.95 | 29.26 | 0.0062 |
| SB03013A2A07.f1 | | | | -1.23 | -2.96 | 29.26 | 0.0060 |
| SB02009B1A07.f1.A | Gga.31032 | CHST1 | Carbohydrate sulfotransferase 1 | -1.23 | -2.98 | 29.26 | 0.0057 |
| SB03006B1B04.f1 | | | | -1.22 | -3.08 | 29.26 | 0.0045 |
| SB03035B2H10.f1 | Gga.35012 | TUBA3D | Tubulin alpha chain | -1.22 | -3.04 | 29.26 | 0.0050 |

| | | | | | | | |
|---|---|---|---|---|---|---|---|
| SB02043A1E08.f1 | Gga.9682 | BCLAF1 | BCL2-associated transcription factor 1 | -1.22 | -3.45 | 29.26 | 0.0017 |
| SB03017B2D12.f1 | Hs.239514 | DGKE | Diacylglycerol kinase, epsilon | -1.22 | -2.77 | 29.26 | 0.0097 |
| SB03028B2B12.f1 | Hs.493771 | C9orf25 | Chromosome 9 open reading frame 25 | -1.21 | -3.02 | 29.26 | 0.0053 |
| SB02026A1C05.f1 | Gga.21932 | TH1L | TH1L Hypothetical protein | -1.21 | -2.79 | 29.26 | 0.0093 |
| SB03022B2B12.f1 | Gga.8053 | TIMM22 | Mitochondrial import inner membrane translocase subunit Tim22 | -1.21 | -3.06 | 29.26 | 0.0047 |
| SB03042A1E07.f1 | Gga.30051 | SGSM3 | Small G protein signaling modulator 3 | -1.21 | -2.93 | 26.26 | 0.0069 |
| SB03018B2E05.f1 | Gga.30898 | PIK3R4 | Phosphoinositide-3-kinase, regulatory subunit 4, p150 | -1.21 | -2.93 | 29.26 | 0.0065 |
| SB02025A1A03.f1 | Gga.2749 | ZNF462 | Zinc finger protein 462 | -1.20 | -2.83 | 29.26 | 0.0083 |
| SB03001A2E08.f1 | Gga.2486 | RAB10 | RAB10, member RAS oncogene family | -1.20 | -2.96 | 29.26 | 0.0060 |
| SB010002000G04 | Gga.4903 | RRN3 | TC190447 + GO | -1.20 | -2.94 | 29.26 | 0.0063 |
| SB02048A2C02.f1 | Gga.7650 | GSK3B | Glycogen synthase kinase 3 beta | -1.20 | -2.76 | 29.26 | 0.0099 |
| SB020001000D12 | Gga.37351 | RNF2 | Ring finger protein 2 | -1.20 | -3.08 | 29.26 | 0.0045 |
| SB02008B2H07.f1 | Gga.12604 | MS4A15 | Membrane-spanning 4-domains, subfamily A, member 15 | -1.20 | -2.88 | 29.26 | 0.0074 |
| SB02034B1F09.f1 | Gga.8044 | PRDX1 | Peroxiredoxin 1 | -1.19 | -3.07 | 29.26 | 0.0046 |
| SB02025B2A10.f1 | | | | -1.19 | -2.76 | 29.26 | 0.0100 |
| SB03018B1E09.f1 | Hs.517262 | SON | SON DNA binding protein | -1.19 | -2.83 | 29.26 | 0.0084 |
| SB010002000B12 | Gga.1342 | KIF5B | kinesin family member 5B | -1.19 | -2.83 | 29.26 | 0.0084 |
| SB03010B1G04.f1 | | | | -1.19 | -2.84 | 29.26 | 0.0081 |
| SB03006B2H08.f1 | Gga.3148 | EXOC5 | Exocyst complex component Sec10 | -1.19 | -3.43 | 29.26 | 0.0018 |
| SB03016B2E11.f1 | Gga.23841 | GNB5 | Guanine nucleotide-binding protein beta subunit 5 | -1.19 | -2.81 | 29.26 | 0.0088 |
| SB03035A2C01.f1 | Gga.1055 | HK2 | Hexokinase 2 | -1.18 | -2.94 | 29.26 | 0.0063 |
| SB02041A1A03.f1 | Gga.22563 | KRR1 | KRR1, small subunit (SSU) processome component | -1.18 | -3.27 | 29.26 | 0.0027 |
| SB02011B1F04.f1 | Gga.31806 | TTC37 | MKIAA0372 protein | -1.18 | -3.06 | 28.26 | 0.0049 |
| SB03012B2G01.f1 | Gga.19307 | GRID2 | Glutamate receptor, ionotropic, delta 2 | -1.18 | -2.80 | 29.26 | 0.0091 |
| SB03008A2F02.f1 | Gga.6358 | C9orf61 | Chromosome 9 open reading frame 61 | -1.18 | -2.83 | 29.26 | 0.0084 |
| SB02048B1A09.f1 | Gga.38986 | TM9SF4 | Transmembrane 9 superfamily protein member 4 | -1.17 | -3.09 | 29.26 | 0.0043 |
| SB03042B2G02.f1 | Gga.19420 | MIER1 | Mesoderm induction early response 1 homolog | -1.17 | -2.79 | 29.26 | 0.0092 |
| SB03007B1C06.f1 | Gga.4601 | LIN7C | Lin-7-C, complete | -1.17 | -3.18 | 29.26 | 0.0035 |
| SB03022B2H04.f1 | Gga.3176 | RNF12 | RING finger protein 12 (LIM domain inter | -1.16 | -2.84 | 29.26 | 0.0082 |
| SB03008B2A01.f1.A | Gga.25133 | ACRC | Acidic repeat containing | -1.16 | -2.92 | 29.26 | 0.0067 |
| SB010019000A02.A | Gga.7325 | GABARAPL2 | Gamma-aminobutyric acid receptor-associated protein like 2 | -1.16 | -2.76 | 29.26 | 0.0100 |
| SB03028A2E03.f1 | Gga.9945 | RBM15B | Putative RNA-binding protein 15B | -1.16 | -2.86 | 29.26 | 0.0078 |
| SB03051B1F05.f1.B.M | | | | -1.15 | -2.76 | 29.26 | 0.0098 |
| SB02022A2H03.f1 | Gga.41286 | EED | Polycomb protein Esc | 1.16 | 2.89 | 29.26 | 0.0072 |
| SB02022B2F07.f1.B | Gga.17987 | JAKMIP2 | Janus kinase and microtubule interacting protein 2 | 1.16 | 3.05 | 29.26 | 0.0048 |
| SB03015A1A12.f1 | Gga.3116 | TRABD | TraB domain containing | 1.17 | 2.93 | 29.26 | 0.0064 |
| SB02003B1D04.f1.B | Gga.2384 | ZFYVE1 | Zinc finger, FYVE domain containing 1 | 1.17 | 3.15 | 29.26 | 0.0038 |
| SB03015A1D03.f1 | Gga.7025 | PSMG2 | Proteasome assembly chaperone 2 | 1.17 | 3.03 | 29.26 | 0.0051 |
| SB03037B2D10.f1 | Gga.4684 | TMEM35 | Splicing factor, proline-and glutamine-rich | 1.17 | 2.89 | 29.26 | 0.0072 |
| SB02041A1A11.f1.B.W | Gga.3243 | CSNK2A1 | Casein kinase 2, alpha 1 polypeptide | 1.18 | 2.79 | 29.26 | 0.0092 |
| SB03009B1B03.f1 | Gga.11342 | GAL3ST2 | Galactose-3-O-sulfotransferase 2 | 1.18 | 3.06 | 28.26 | 0.0048 |
| SB03006A1H08.f1 | Gga.17033 | ARHGEF6 | Rho guanine nucleotide exchange factor 6 | 1.18 | 3.48 | 29.26 | 0.0016 |
| SB02027A2G05.f1 | Gga.28057 | GTF2F2 | Transcription initiation factor IIF beta subunit | 1.18 | 3.47 | 29.26 | 0.0016 |
| SB02012A2F10.f1.B | Gga.5473 | CHCHD10 | Coiled-coil-helix-coiled-coil-helix doma | 1.19 | 3.13 | 29.26 | 0.0040 |
| SB02043A2G01.f1 | Gga.12366 | NUP37 | Nucleoporin Nup37 | 1.19 | 2.79 | 29.26 | 0.0092 |
| SB02038B1A07.f1 | Gga.2904 | SNUPN | Snurportin 1 | 1.19 | 3.18 | 29.26 | 0.0035 |
| SB03001A1G05.f1 | Gga.8084 | TMEM126A | Transmembrane protein 126A | 1.20 | 3.80 | 29.26 | 0.0007 |
| SB02016B2D05.f1 | | | | 1.20 | 3.29 | 29.26 | 0.0026 |
| SB02023B2G09.f1 | Gga.31156 | C10orf137 | Chromosome 16 open reading frame 73 | 1.20 | 3.12 | 29.26 | 0.0041 |
| SB03030B1F09.f1 | Gga.34543 | CSNK1A1 | Casein kinase I, alpha isoform | 1.20 | 3.16 | 29.26 | 0.0036 |
| SB02022A2D12.f1 | Gga.12569 | FNBP1L | Formin binding protein 1-like | 1.20 | 3.87 | 29.26 | 0.0006 |
| SB02049B1E09.f1 | Gga.18098 | LARP1 | La-related protein 1 | 1.20 | 2.82 | 29.26 | 0.0085 |
| SB010001001F11 | Gga.7860 | XPO6 | Exportin 6 | 1.21 | 2.94 | 29.26 | 0.0064 |
| SB02022B2B04.f1 | | | | 1.21 | 2.76 | 29.26 | 0.0098 |
| SB03005A2E08.f1 | Gga.39652 | DNALI1 | Dynein, axonemal, light intermediate chain 1 | 1.21 | 2.87 | 29.26 | 0.0075 |
| SB02008A2F06.f1 | Gga.6296 | SAR1B | GTP-binding protein SAR1b | 1.21 | 2.96 | 29.26 | 0.0061 |
| SB02011B2D01.f1.B.W | Gga.27429 | ABI2 | Abl-interactor 2 | 1.21 | 2.78 | 29.26 | 0.0094 |
| SB02025A2H03.f1.B.W | Gga.13432 | R3HDM1 | R3H domain protein 1 | 1.22 | 3.28 | 29.26 | 0.0027 |
| SB02031A2E05.f1 | Gga.9866 | RALB | V-ral simian leukemia viral oncogene homolog B | 1.22 | 3.31 | 29.26 | 0.0025 |
| SB03032B2A10.f1.A | | | | 1.22 | 2.85 | 29.26 | 0.0079 |
| SB03029A1A12.f1 | Gga.42914 | HS3ST6 | Heparan sulfate glucosamine 3-O-sulfotransferase 6 | 1.23 | 3.12 | 29.26 | 0.0040 |
| SB02012A1E02.f1 | Hs.375837 | C10orf93 | Putative uncharacterized protein C10orf93 | 1.23 | 2.87 | 29.26 | 0.0076 |
| SB02005A2F01.f1.B | Gga.26588 | DLG3 | Disks large homolog 3 | 1.23 | 2.87 | 29.26 | 0.0076 |
| SB010018000H11 | Gga.883 | RFC2 | Activator 1 40 kDa subunit | 1.23 | 3.26 | 29.26 | 0.0028 |
| SB03046A1B11.f1 | Gga.208 | WNT7A | Wingless-type MMTV integration site family, member 7A | 1.23 | 2.84 | 29.26 | 0.0081 |
| SB02028B2C12.f1 | Gga.9706 | CHMP2B | Charged multivesicular body protein 2b | 1.23 | 3.84 | 29.26 | 0.0006 |
| SB03045A1D10.f1 | Gga.21092 | PHF20L1 | PHD finger protein 20-like 1 | 1.23 | 3.20 | 29.26 | 0.0033 |
| SB03039A1D05.f1 | Hs.185489 | LYRM1 | LYR motif-containing protein 1 | 1.23 | 2.90 | 29.26 | 0.0070 |
| SB02049A1H06.f1.B | Gga.40030 | ZFYVE9 | Zinc finger FYVE domain-containing protein 9 | 1.24 | 3.36 | 29.26 | 0.0022 |
| SB02040B2D04.f1 | Gga.37954 | CHKA | Choline kinase alpha | 1.24 | 3.62 | 29.26 | 0.0011 |
| SB03023A2B06.f1 | Gga.1184 | PSMB4 | Proteasome subunit beta type 4 precursor | 1.24 | 3.88 | 29.26 | 0.0005 |
| SB03042A2C12.f1 | Hs.445574 | C17orf96 | Chromosome 17 open reading frame 96 | 1.24 | 2.81 | 28.26 | 0.0089 |
| SB03028B1D02.f1 | Gga.42380 | AMFR | Autocrine motility factor receptor | 1.24 | 3.43 | 29.26 | 0.0018 |
| SB03007B2H01.f1 | Hs.551747 | NEU4 | Sialidase 4 | 1.24 | 2.85 | 29.26 | 0.0080 |
| SB02040B2C06.f1 | Gga.7158 | CD164 | Probable aspartate aminotransferase | 1.24 | 2.89 | 29.26 | 0.0072 |
| SB03024B2A05.f1 | Gga.9061 | RTCD1 | RNA 3'-terminal phosphate cyclase | 1.24 | 2.99 | 29.26 | 0.0056 |
| SB02027A2B12.f1 | Gga.2833 | SNF1LK | Qin-induced kinase, complete | 1.24 | 3.02 | 29.26 | 0.0052 |
| SB02027A2B05.f1 | Gga.15424 | WDSOF1 | WD repeats and SOF1 domain containing | 1.24 | 3.04 | 29.26 | 0.0050 |
| SB02009B1E10.f1 | Gga.12452 | ITGB1BP1 | Integrin beta-1 binding protein 1 | 1.24 | 2.85 | 29.26 | 0.0079 |
| SB03003A2A10.f1 | Gga.30817 | RNF144A | C3HC4 zinc-finger in the C-termina | 1.24 | 2.99 | 29.26 | 0.0057 |
| SB02032B1H06.f2.B | Gga.7793 | ADAM17 | ADAM metallopeptidase domain 17 | 1.25 | 2.81 | 29.26 | 0.0088 |
| SB02037A2H12.f1 | Gga.2751 | UQCRQ | Ubiquinol-cytochrome c reductase complex | 1.25 | 3.20 | 29.26 | 0.0033 |
| SB010014000H04 | Gga.3831 | PACSIN2 | Protein kinase C and casein kinase substrate in neurons protein 2 | 1.25 | 3.17 | 29.26 | 0.0036 |
| SB02043B1A05.f1 | Gga.17148 | UBE3A | Ubiquitin protein ligase E3A | 1.25 | 2.92 | 29.26 | 0.0067 |

| | | | | | | | |
|---|---|---|---|---|---|---|---|
| SB02014B1A04.f1 | Gga.768 | TGIF1 | Homeobox protein AKR | 1.25 | 3.08 | 29.26 | 0.0045 |
| SB03005B2A03.f1 | Gga.8061 | HSCB | Similar to J-type co-chaperone HSC20 | 1.25 | 3.01 | 29.26 | 0.0053 |
| SB03040A2C10.f1.B | Gga.537 | VEGFA | Vascular endothelial growth factor A Precursor | 1.25 | 3.81 | 29.26 | 0.0007 |
| SB02022A1G04.f1 | | | | 1.25 | 2.91 | 29.26 | 0.0068 |
| SB03043A1C12.f1.B | Gga.24614 | TSPAN10 | Tetraspanin 10 | 1.25 | 3.10 | 29.26 | 0.0042 |
| SB02005A2F09.f1.B | | | | 1.26 | 3.03 | 29.26 | 0.0051 |
| SB02042A2C07.f1 | Gga.9785 | NMT2 | N-myristoyltransferase 2 | 1.26 | 3.71 | 29.26 | 0.0009 |
| SB03027B2F02.f1 | Gga.34340 | NRN1 | Neuritin 1 | 1.26 | 3.12 | 29.26 | 0.0040 |
| SB03003B2B03.f1 | Gga.8480 | HMBS | Porphobilinogen deaminase | 1.26 | 3.34 | 29.26 | 0.0023 |
| SB03043A1B03.f1 | Gga.2718 | NUDT14 | Nudix (nucleoside diphosphate linked moiety X)-type motif 14 | 1.26 | 2.96 | 29.26 | 0.0061 |
| SB010009001H01 | Gga.3222 | LIMK2 | LIM domain kinase 2 | 1.26 | 3.37 | 29.26 | 0.0021 |
| SB03047B2H12.f1 | Gga.3406 | FOXO1 | Forkhead protein FKHR | 1.26 | 2.79 | 29.26 | 0.0091 |
| SB02003A1B05.f2 | Hs.387856 | TTC28 | Tetratricopeptide repeat domain 28 | 1.27 | 2.81 | 29.26 | 0.0089 |
| SB02031A1E06.f1 | Gga.8788 | SLC25A29 | Solute carrier family 25, member 29 | 1.27 | 3.44 | 29.26 | 0.0018 |
| SB02009A1F10.f1 | Gga.12450 | GATAD1 | Ocular development-associated gene | 1.27 | 3.22 | 29.26 | 0.0031 |
| SB02014B1E05.f1 | Gga.8084 | TMEM126A | Transmembrane protein 126A | 1.27 | 3.56 | 29.26 | 0.0013 |
| SB02014A1B06.f2 | Gga.31705 | GLCCI1 | Glucocorticoid induced transcript 1 | 1.27 | 3.59 | 29.26 | 0.0012 |
| SB010021000C09 | Hs.709353 | CACNB2 | Calcium channel, voltage-dependent, beta 2 subunit | 1.27 | 2.79 | 29.26 | 0.0092 |
| SB02012A2G08.f1 | Gga.20086 | ACAT2 | Acetyl-CoA acetyltransferase | 1.27 | 3.07 | 29.26 | 0.0046 |
| SB02042A2F04.f1.A | Gga.3071 | SELS | Selenoprotein S | 1.27 | 2.83 | 29.26 | 0.0083 |
| SB03046B1A10.f1 | | | | 1.28 | 2.76 | 29.26 | 0.0099 |
| SB02004B1H07.f1 | Gga.21366 | HSPH1 | Heat-shock protein 105 kDa | 1.28 | 3.55 | 29.26 | 0.0013 |
| SB010004001B01 | Gga.9053 | XBP1 | X-box binding protein 1 | 1.28 | 3.09 | 29.26 | 0.0044 |
| SB010015000F04 | Gga.12237 | STX18 | Syntaxin-18 | 1.28 | 2.91 | 29.26 | 0.0068 |
| SB02014A2B07.f2 | Gga.5690 | RBM24 | RNA-binding region | 1.28 | 2.83 | 28.26 | 0.0085 |
| SB02003B1G07.f1 | Gga.9995 | CROT | similar to carnitine octanoyltransferase | 1.28 | 2.85 | 29.26 | 0.0080 |
| SB010004001E05 | Gga.5754 | SUZ12 | Suppressor of zeste 12 homolog | 1.28 | 3.07 | 29.26 | 0.0045 |
| SB02004A2D01.f1.B | Gga.3562 | MRPL12 | 39S ribosomal protein L12, mitochondrial | 1.29 | 3.63 | 29.26 | 0.0011 |
| SB02015B1F07.f1 | Gga.27627 | TIAM2 | Sif and Tiam1-like exchange factor | 1.29 | 2.86 | 29.26 | 0.0077 |
| SB02049A1A02.f1 | Gga.8606 | PGK2 | Phosphoglycerate kinase | 1.29 | 3.05 | 29.26 | 0.0048 |
| SB010010000H04 | Gga.41559 | LRRC38 | Leucine rich repeat containing 38 | 1.29 | 2.94 | 29.26 | 0.0064 |
| SB03041B1A02.f1.B | Gga.10026 | VPS37B | hypothetical protein FLJ12750 | 1.30 | 3.39 | 29.26 | 0.0020 |
| SB03048B1D04.f1 | Gga.22064 | THAP5 | THAP domain containing 5 | 1.30 | 3.01 | 29.26 | 0.0053 |
| SB03020A2D11.f1 | Gga.10624 | ZBTB41 | Zinc finger protein 226 | 1.30 | 3.04 | 29.26 | 0.0050 |
| SB03021A1D05.f1 | Gga.39100 | LRWD1 | Leucine-rich repeats and WD repeat domain containing 1 | 1.30 | 2.79 | 29.26 | 0.0092 |
| SB03015B1E05.f1 | | | | 1.30 | 3.77 | 29.26 | 0.0007 |
| SB02009B2G05.f1.B | Gga.22402 | COMMD10 | COMM domain containing protein 10 | 1.30 | 3.67 | 29.26 | 0.0010 |
| SB03041B2G04.f1 | Gga.28695 | AGTPBP1 | ATP/GTP binding protein 1 | 1.31 | 2.86 | 29.26 | 0.0078 |
| SB03009A1D07.f1 | | | | 1.31 | 3.02 | 29.26 | 0.0052 |
| SB03024B2F12.f1 | Gga.3323 | FABP5 | Fatty acid binding protein 5 | 1.31 | 3.04 | 29.26 | 0.0049 |
| SB03031A2G05.f1 | Gga.15373 | DDX31 | Probable ATP-dependent RNA helicase DDX31 | 1.31 | 3.20 | 29.26 | 0.0033 |
| SB02017B1G09.f1 | | | | 1.31 | 2.83 | 29.26 | 0.0084 |
| SB02027A1H01.f1 | Gga.2736 | ORC2L | Origin recognition complex, subunit 2-like | 1.32 | 2.80 | 29.26 | 0.0091 |
| SB03026B1H09.f1 | Gga.150 | FLT1 | Fms-related tyrosine kinase 1 | 1.32 | 3.39 | 29.26 | 0.0020 |
| SB02017B1H06.f1 | Gga.12894 | DNAJB4 | DnaJ (Hsp40) homolog, subfamily B, member 4 | 1.32 | 2.78 | 29.26 | 0.0093 |
| SB02029A1B08.f1 | Gga.40185 | NOD1 | Nucleotide-binding oligomerization domain containing 1 | 1.32 | 3.25 | 29.26 | 0.0029 |
| SB02011A2E09.f1 | Gga.3680 | SEPT2 | Septin 2 (NEDD5 protein homolog) | 1.32 | 3.22 | 29.26 | 0.0032 |
| SB02019B2D05.f1 | Gga.30082 | TAF5L | TAF5-like RNA polymerase II p300/CBP-associated factor 65 kDa subunit 5L | 1.32 | 3.05 | 29.26 | 0.0049 |
| SB02015A1G08.f1 | Gga.4766 | POLR2D | DNA-directed RNA polymerase II 16 kDa | 1.33 | 3.18 | 29.26 | 0.0035 |
| SB02023A1D07.f1 | Gga.5884 | DYNLL1 | Dynein, cytoplasmic, light chain 1 | 1.33 | 3.24 | 29.26 | 0.0030 |
| SB02042A1A07.f1 | | | | 1.33 | 3.52 | 29.26 | 0.0015 |
| SB010015000A11 | | | | 1.33 | 3.08 | 29.26 | 0.0044 |
| SB03023B2G08.f1 | | | | 1.33 | 2.77 | 29.26 | 0.0096 |
| SB02019A2D07.f1 | Gga.40345 | ENTPD3 | Ectonucleoside triphosphate diphosphohydrolase 3 | 1.33 | 2.80 | 29.26 | 0.0090 |
| SB02017B1D07.f1 | | | | 1.34 | 2.90 | 29.26 | 0.0071 |
| SB03010B1F10.f1 | Gga.3178 | DACH2 | Dachshund homolog 2 | 1.34 | 2.93 | 29.26 | 0.0065 |
| SB02013A2A11.f1 | Gga.36586 | ADCK2 | AarF domain containing kinase 2, partial | 1.34 | 2.82 | 29.26 | 0.0085 |
| SB02034A2H05.f1 | Gga.40378 | PITPNC1 | Phosphatidylinositol transfer protein, cytoplasmic 1 | 1.35 | 3.07 | 29.26 | 0.0046 |
| SB02010B1D10.f1 | Gga.30233 | RPS6KA2 | Ribosomal protein S6 kinase, polypeptide 2 | 1.37 | 3.95 | 29.26 | 0.0005 |
| SB02033B2G04.f1 | Gga.28057 | GTF2F2 | General transcription factor IIF, polypeptide 2, 30kDa | 1.37 | 3.95 | 29.26 | 0.0005 |
| SB03027A1C08.f1.M | Gga.14002 | DENND4C | DENN/MADD domain containing 4C | 1.37 | 3.22 | 29.26 | 0.0031 |
| SB03003A1F12.f1 | Gga.44589 | ITGA6 | Integrin, alpha 6 | 1.39 | 3.67 | 29.26 | 0.0010 |
| SB03030B2B09.f1 | Gga.23837 | FBXL2 | F-box/LRR-repeat protein 2 | 1.39 | 2.97 | 29.26 | 0.0059 |
| SB03044B1B10.f1 | Gga.5900 | DNAJA1 | similar to pDJA1 chaperone | 1.40 | 5.09 | 29.26 | 0.0000 |
| SB03049A2F11.f1 | Gga.16800 | MAPRE1 | Microtubule-associated protein RP/EB family member 1 | 1.41 | 4.01 | 29.26 | 0.0004 |
| SB03048A2G09.f1 | | | | 1.41 | 3.45 | 29.26 | 0.0017 |
| SB03002B2H04.f1 | | | | 1.42 | 2.98 | 29.26 | 0.0058 |
| SB02031A2C06.f1 | | | | 1.42 | 3.96 | 29.26 | 0.0004 |
| SB02046B2E09.f1 | Hs.308710 | JHDM1D | Jumonji C domain-containing histone demethylase 1 homolog D | 1.42 | 2.94 | 29.26 | 0.0064 |
| SB02020B1G02.f1 | Gga.12894 | DNAJB4 | DnaJ (Hsp40) homolog, subfamily B, member 4 | 1.43 | 3.72 | 29.26 | 0.0008 |
| SB02038A1C02.f1 | Gga.11181 | BACH2 | BTB and CNC homology 1, basic leucine zipper transcription factor 2 | 1.44 | 3.44 | 29.26 | 0.0018 |
| SB03019A1H01.f1 | Gga.19165 | VRK2 | Serine/threonine-protein kinase VRK2 | 1.45 | 3.17 | 29.26 | 0.0035 |
| SB03022A2C01.f1 | Gga.9158 | AHSA2 | Activator of heat shock 90kDa protein ATPase homolog 2 | 1.45 | 5.15 | 29.26 | 0.0000 |
| SB02038B1F08.f1 | Gga.28057 | GTF2F2 | General transcription factor IIF, polypeptide 2, 30kDa | 1.46 | 3.08 | 29.26 | 0.0045 |
| SB03024B1A12.f1 | Gga.32314 | SLC38A4 | Solute carrier family 38, member 4 | 1.48 | 3.04 | 29.26 | 0.0049 |
| SB03033A2C05.f1 | Gga.30133 | NR4A3 | Orphan nuclear receptor NR4A3 | 1.49 | 3.33 | 29.26 | 0.0024 |
| SB02029A1F03.f1 | | | | 1.50 | 2.79 | 29.26 | 0.0091 |
| SB02025A1C03.f1 | Gga.5799 | P4HA2 | Prolyl 4-hydroxylase alpha-2 subunit precursor | 1.56 | 2.95 | 29.26 | 0.0062 |
| SB02034A1F12.f1.B.W | Gga.38109 | MGST1 | Microsomal glutathione S-transferase 1 | 1.56 | 2.95 | 29.26 | 0.0062 |
| SB03044A2D03.f1 | Hs.719230 | HSPA2 | Heat shock 70 kDa protein 2 | 1.57 | 3.38 | 29.26 | 0.0021 |
| SB03009B2H04.f1 | Gga.5799 | P4HA2 | Prolyl 4-hydroxylase alpha-2 subunit precursor | 1.69 | 4.31 | 29.26 | 0.0002 |
| SB03046A2H10.f1 | Hs.719374 | OPA3 | Optic atrophy 3 protein | 1.73 | 4.69 | 29.26 | 0.0001 |
| SB03037B2H05.f1 | Hs.719230 | HSPA2 | Heat shock 70 kDa protein 2 | 1.76 | 4.68 | 29.26 | 0.0001 |
| SB03032B2H11.f1 | | | | 1.76 | 3.11 | 29.26 | 0.0042 |

| | | | | | | | |
|---|---|---|---|---|---|---|---|
| SB02032A2C07.f2 | Gga.30876 | DMAP1 | DNA methyltransferase 1 associated protein 1 | 1.94 | 2.87 | 29.26 | 0.0075 |
| SB02021A2E01.f1 | Gga.405 | ITPKA | Inositol 1,4,5-trisphosphate 3-kinase A | 2.07 | 3.69 | 29.26 | 0.0009 |
| SB010015000G04 | | | | 2.34 | 3.43 | 29.26 | 0.0018 |
| SB02004A2D02.f1 | Gga.27948 | C10orf56 | chromosome 10 open reading frame 56 | 3.41 | 2.88 | 29.26 | 0.0073 |
| SB02003A1C05.f2.B | Gga.11856 | NRSN1 | Neurensin 1 | 4.21 | 2.91 | 29.26 | 0.0069 |
| SB02032A1H02.f2.B | Gga.28740 | C17orf56 | Chromosome 17 open reading frame 56 | 5.17 | 2.82 | 28.26 | 0.0088 |
| SB02043A2F01.f1.B | Gga.16557 | ARL5A | ADP-ribosylation factor-like protein 5 | 6.80 | 3.03 | 28.26 | 0.0052 |
| SB03038B1F04.f1 | Gga.2620 | TTR | Transthyretin precursor | 8.34 | 3.00 | 29.26 | 0.0055 |
| SB03035A2H04.f1.B | Gga.41728 | LBXCOR1 | Ladybird homeobox corepressor 1 | 8.50 | 3.07 | 29.26 | 0.0046 |
| SB02032B2F01.f2 | Gga.2620 | TTR | Transthyretin precursor | 9.84 | 3.12 | 29.26 | 0.0040 |
| SB02026B2H07.f1 | Gga.2620 | TTR | Transthyretin precursor | 9.97 | 3.07 | 29.26 | 0.0045 |
| SB02015A2G08.f1 | Gga.2620 | TTR | Transthyretin precursor | 13.34 | 3.33 | 29.26 | 0.0024 |
