# Supplementary material for: Seasonal Differences of Gene Expression Profiles in Song Sparrow (Melospiza melodia) Hypothalamus in Relation to Territorial Aggression
Source: PLoS One. 2009 Dec 4;4(12):e8182. doi: 10.1371/journal.pone.0008182 (PMC2780717; doi:10.1371/journal.pone.0008182)
Supplement: Table S2 — Complete list of cDNAs affected by the comparison AE vs. SE with cell-means model, p<0.01. The expressions in autumn STI (AE) compared to spring STI (SE) are shown in fold changes. (0.02 MB PDF) [file pone.0008182.s002.pdf]

Spot ID	UniGene ID	HGNC_symbol	Gene Description	Fold Change AE vs. SE	T Statistic	df	P-value
SB02006A2H05.f1	Gga.28520	CGA	Glycoprotein hormones alpha chain precursor	-7.43	-7.67	29.26	0.0000
SB03032A2G07.f1	Gga.1079	CRYM	Mu-crystallin homolog	-2.40	-3.84	29.26	0.0006
SB03047B2E11.f1	Gga.1079	CRYM	Mu-crystallin homolog	-2.31	-3.94	29.26	0.0005
SB02022A2D01.f1	Gga.11726	GADD45B	Growth arrest and DNA-damage-inducible, beta	-2.26	-5.70	29.26	0.0000
SB03046A2G09.f1				-2.21	-3.26	29.26	0.0028
SB03046B1C04.f1	Gga.1079	CRYM	Mu-crystallin homolog	-2.05	-3.59	29.26	0.0012
SB03028B1F05.f1				-1.98	-2.79	29.26	0.0091
SB02005B2G07.f1				-1.91	-2.79	29.26	0.0092
SB02011A1B09.f1	Gga.11430	PENK	Proenkephalin A Precursor	-1.70	-3.20	29.26	0.0033
SB020001000D07	Gga.11430	PENK	Proenkephalin A Precursor	-1.65	-3.55	29.26	0.0013
SB02025A2E07.f1				-1.64	-2.81	29.26	0.0087
SB010006000E12				-1.63	-4.07	29.26	0.0003
SB02007A1D12.f1	Gga.38496	SMTNL1	Smoothelin-like protein 1	-1.63	-5.02	29.26	0.0000
SB03030B2C09.f1	Gga.28278	C5orf40	chromosome 5 open reading frame 40	-1.62	-2.92	29.26	0.0067
SB03046A2A02.f1	Gga.21371	NIT2	Nitrilase homolog 2	-1.60	-3.21	29.26	0.0032
SB010015000A05	Gga.28278	C5orf40	chromosome 5 open reading frame 40	-1.60	-3.12	29.26	0.0040
SB03042B1B07.f1	Gga.39788	GREB1	similar to growth regulation by estrogen in breast cancer protein	-1.57	-4.37	28.26	0.0002
SB03034B2D05.f1	Gga.17584	ZBTB16	Zinc finger and BTB domain-containing protein 16	-1.55	-3.55	29.26	0.0013
SB02023B2D11.f1	Gga.4974	VCAN	Versican core protein Precursor	-1.54	-3.91	29.26	0.0005
SB03006A1B07.f1	Gga.944	CENPH	Centromere protein H	-1.54	-3.03	29.26	0.0051
SB010013001H09	Gga.3168	QKI	Protein quaking (HqkI)	-1.52	-4.16	29.26	0.0003
SB010020000D02.B	Gga.14972	LRRC3B	Leucine-rich repeat-containing protein 3B Precursor	-1.51	-3.25	29.26	0.0029
SB02027A2C02.f1	Gga.40320	GLP2R	Glucagon-like peptide 2 receptor Precursor	-1.49	-3.09	29.26	0.0043
SB03021A1B12.f1	Gga.21228	ABCG1	ATP-binding cassette sub-family G member 1	-1.48	-4.66	29.26	0.0001
SB03033A1H12.f1				-1.47	-3.26	29.26	0.0028
SB03048A2E10.f1	Gga.31071	CEP78	Centrosomal protein of 78 kDa	-1.46	-3.91	29.26	0.0005
SB02037B1A04.f1	Gga.25837	ADAMTS19	ADAM metallopeptidase with thrombospondin type 1 motif, 19	-1.46	-4.42	29.26	0.0001
SB03050A1E08.f1	Gga.3370	OCIAD2	OCIA domain-containing protein 2	-1.45	-3.17	29.26	0.0036
SB03043A1F02.f1	Gga.102	ABCA1	ATP-binding cassette sub-family A member 1	-1.45	-4.08	29.26	0.0003
SB02009A2E02.f1	Gga.2764	SLC35F1	Solute carrier family 35 member F1	-1.44	-2.92	29.26	0.0068
SB03036B1C02.f1	Gga.17584	ZBTB16	Zinc finger and BTB domain-containing protein 16	-1.42	-3.82	29.26	0.0006
SB03043B1E08.f1	Gga.2937	NFKBIA	NF-kappa-B inhibitor alpha	-1.42	-3.10	29.26	0.0043
SB02034B1H05.f1	Gga.15742	LARP4	La-related protein 4	-1.41	-3.43	28.26	0.0019
SB03005A2H09.f1	Gga.20550	ABCA1	ATP-binding cassette sub-family A member 1	-1.40	-3.39	29.26	0.0020
SB02035A1G11.f1	Gga.9908	MRPL50	39S ribosomal protein L50, mitochondrial	-1.39	-2.94	29.26	0.0064
SB03041A2C02.f1	Gga.5899	FAM125B	Protein FAM125B	-1.38	-3.09	29.26	0.0044
SB02011B1D08.f1	Gga.1210	CCRN4L	carbon catabolite repression 4-like	-1.37	-3.79	29.26	0.0007
SB010022000E01				-1.37	-3.17	29.26	0.0036
SB03002B1B06.f1	Gga.7347	MCAT	Malonyl CoA-acyl carrier protein transacylase, mitochondrial Precursor	-1.37	-2.99	29.26	0.0056
SB03042A2C10.f1	Gga.4941	CDH11	Cadherin-11 Precursor	-1.37	-2.95	29.26	0.0063
SB03034B2G06.f1	Gga.12295	PPAPR3	Lipid phosphate phosphatase-related protein type 1	-1.37	-2.82	29.26	0.0086
SB02020A2B09.f1	Gga.7482	SETD4	SET domain-containing protein 4	-1.36	-2.92	29.26	0.0067
SB010010000G09	Gga.10902	RASL11A	Ras-like protein family member 11A	-1.36	-2.80	29.26	0.0090
SB03007B1E09.f1	Gga.20327	KLF13	Krueppel-like factor 13	-1.36	-3.15	29.26	0.0038
SB03028A2E06.f1	Gga.29845	RBM15	Putative RNA-binding protein 15	-1.35	-5.48	29.26	0.0000
SB02031B2F04.f1				-1.34	-3.41	29.26	0.0019
SB02033B1G10.f1	Gga.9668	ATRX	Transcriptional regulator ATRX	-1.34	-3.10	28.26	0.0044
SB03035A1E08.f1	Gga.25176	TTC19	Tetratricopeptide repeat protein 19	-1.32	-4.16	29.26	0.0003
SB02009B2F04.f1	Gga.5775	HNRNPA3	Heterogeneous nuclear ribonucleoprotein A3 (hnRNP A3)	-1.32	-6.22	29.26	0.0000
SB03008A2B06.f1	Gga.6508	GLRA2	Glycine receptor subunit alpha-2 Precursor	-1.32	-3.11	29.26	0.0041
SB02031A1F10.f1	Gga.31071	CEP78	Centrosomal protein of 78 kDa	-1.32	-3.03	29.26	0.0051
SB03011B1B10.f1	Gga.9774	RBM9	RNA-binding motif protein 9	-1.32	-2.95	29.26	0.0062
SB02049B1H01.f1	Gga.41617	NTF3	Neurotrophin-3 Precursor	-1.32	-3.45	29.26	0.0017
SB010009001B06	Gga.30217	ESCO1	N-acetyltransferase ESCO1	-1.31	-3.57	29.26	0.0013
SB03004A2B07.f1				-1.31	-2.96	29.26	0.0060
SB02043B1F05.f1				-1.31	-3.20	29.26	0.0033
SB02027B2B09.f1				-1.31	-2.97	29.26	0.0059
SB02016A2C01.f1	Gga.10504	CASKIN2	Caskin-2	-1.30	-3.14	28.26	0.0040
SB02040A2H01.f1	Gga.39911	ZGPAT	Zinc finger CCCH-type with G patch domain-containing protein	-1.30	-3.04	29.26	0.0049
SB03045A2D08.f1				-1.30	-3.69	29.26	0.0009
SB03025B1E03.f2	Gga.5829	PSME4	Proteasome activator complex subunit 4	-1.30	-3.21	29.26	0.0032
SB010003000B02.B				-1.30	-3.01	29.26	0.0054
SB03038B2F09.f1	Gga.3091	SCN5A	Sodium channel protein type 5 subunit alpha	-1.29	-2.92	29.26	0.0067
SB03009B1B05.f1	Gga.31229	SCN1A	Sodium channel protein type 1 subunit alpha	-1.29	-3.08	29.26	0.0045
SB03042A2H10.f1	Gga.12278	ADAT2	tRNA-specific adenosine deaminase 2	-1.29	-2.79	29.26	0.0092
SB03009B1B02.f1	Gga.22426	KIAA1370	Uncharacterized protein KIAA1370	-1.29	-3.42	29.26	0.0019
SB03019A2H03.f1				-1.29	-3.00	29.26	0.0054
SB03009A2A03.f1	Gga.10477	TNFRSF19	Tumor necrosis factor receptor superfamily member 19 Precursor	-1.28	-2.82	29.26	0.0085
SB03008A1D08.f1	Gga.10043	PICK1	Protein interacting with C kinase 1	-1.28	-3.52	29.26	0.0014
SB010024000A05				-1.28	-3.23	29.26	0.0031
SB03015A2A11.f1.A				-1.28	-2.80	29.26	0.0091
SB03020A1C12.f1	Hs.651939	MAGI1	Membrane associated guanylate kinase, WW and PDZ domain containing 1	-1.28	-2.84	29.26	0.0080
SB02020A2B05.f1.B				-1.28	-3.23	29.26	0.0031
SB03049B1H03.f1	Gga.19496	C1orf114	Uncharacterized protein C1orf114	-1.27	-2.83	29.26	0.0083
SB03041B2E04.f1	Gga.36295	C16orf45	Uncharacterized protein C16orf45	-1.27	-3.10	29.26	0.0043
SB03008B2C03.f1				-1.27	-3.52	29.26	0.0014
SB02010A2C05.f1	Gga.8061	HSCB	Co-chaperone protein HscB, mitochondrial Precursor	-1.26	-3.47	29.26	0.0016
SB02008B2H04.f1	Gga.27391	C9orf6	chromosome 9 open reading frame 6	-1.26	-3.35	29.26	0.0022
SB03039B1G07.f1	Gga.43357	RAC3	Ras-related C3 botulinum toxin substrate 3 Precursor	-1.26	-2.77	29.26	0.0096
SB03003B2D06.f1	Gga.19997	CITED2	Cbp/p300-interacting transactivator 2	-1.26	-3.38	29.26	0.0021
SB03046A2C01.f1	Gga.13455	DYRK2	Dual specificity tyrosine-phosphorylation-regulated kinase 2	-1.25	-2.97	29.26	0.0059
SB02036B1C11.f1	Gga.34693	PION	Protein pigeon homolog	-1.25	-2.85	29.26	0.0079
SB02029B1D08.f1	Hs.348262	KLHL13	Kelch-like protein 13 (BTB and kelch domain-containing protein 2)	-1.25	-3.79	29.26	0.0007
SB02030A2A11.f1	Gga.26601	AP1S2	AP-1 complex subunit sigma-2	-1.25	-2.89	29.26	0.0072
SB02016B1C07.f1	Gga.9325	UBE2T	Ubiquitin-conjugating enzyme E2 T	-1.25	-3.61	29.26	0.0011
SB010004001E12.B				-1.25	-2.77	29.26	0.0097

SB02014A1D08.f2				-1.25	-3.24	29.26	0.0030
SB03002B1A11.f1	Gga.29908	ST3GAL5	Lactosylceramide alpha-2,3-sialyltransferase	-1.25	-2.97	29.26	0.0059
SB02034A1D09.f1	Gga.2646	SLC33A1	Acetyl-coenzyme A transporter 1 (Solute carrier family 33 member 1)	-1.25	-3.15	29.26	0.0037
SB03044B1H02.f1	Gga.14699	SH3D19	SH3 domain-containing protein 19	-1.25	-2.78	29.26	0.0094
SB03033B1H08.f1	Gga.28477	PAPD5	PAP associated domain containing 5 isoform a	-1.25	-3.51	29.26	0.0015
SB03002A1G04.f1				-1.24	-3.59	29.26	0.0012
SB010021000E01	Gga.10727	PAPSS1	Bifunctional 3'-phosphoadenosine 5'-phosphosulfate synthetase 1	-1.24	-2.99	29.26	0.0057
SB02022A1D03.f1	Gga.17666	FNBP1	Formin-binding protein 1	-1.24	-2.88	29.26	0.0073
SB03028B1C10.f1.A	Gga.1042	RPS10	40S ribosomal protein S10	-1.24	-3.68	29.26	0.0009
SB02025A1A03.f1	Gga.1156	ZNF462	Zinc finger protein 462	-1.24	-3.36	29.26	0.0022
SB02020A2B05.f1.A				-1.23	-2.93	29.26	0.0066
SB03020A1A03.f1	Gga.22508	C6orf130	Uncharacterized protein C6orf130	-1.23	-2.83	29.26	0.0084
SB03021B1A10.f1	Gga.17597	MSI2	RNA-binding protein Musashi homolog 2	-1.23	-3.09	29.26	0.0044
SB02014B1C05.f1	Gga.17315	C12orf51-1	Transmembrane protein C12orf51	-1.23	-3.21	28.26	0.0033
SB03022B1E10.f1	Gga.29151	OTUD7A	OTU domain-containing protein 7A	-1.23	-2.82	29.26	0.0085
SB03031A1E09.f1	Gga.22503	EXOC6	Exocyst complex component 6	-1.23	-2.92	29.26	0.0067
SB03014B1B02.f1	Gga.30662	PEX13	Peroxisomal membrane protein PEX13 (Peroxin-13)	-1.22	-3.59	28.26	0.0012
SB03051A2H05.f1	Gga.24279	RASGEF1B	Ras-GEF domain-containing family member 1B	-1.22	-2.79	29.26	0.0092
SB02018A2D06.f1	Gga.22733	ALKBH1	Alkylated DNA repair protein alkB homolog 1	-1.22	-2.81	29.26	0.0087
SB03034A2A04.f1.M				-1.22	-3.58	29.26	0.0012
SB02011B1F04.f1	Gga.31806	TTC37	Tetratricopeptide repeat protein 37	-1.22	-3.71	28.26	0.0009
SB03016A2G07.f1	Gga.5964	UROS	Uroporphyrinogen-III synthase	-1.22	-3.07	29.26	0.0046
SB03020A1G07.f1	Gga.22540	USP1	Ubiquitin carboxyl-terminal hydrolase 1	-1.21	-2.93	27.26	0.0068
SB03011B1D06.f1	Gga.11639	XPR1	Xenotropic and polytropic retrovirus receptor 1	-1.21	-2.77	29.26	0.0097
SB03018A2F08.f1	Gga.12924	SGCE	sarcoglycan, epsilon	-1.21	-3.25	29.26	0.0029
SB02036A2C06.f1	Gga.19224	ZXDC	ZXD family zinc finger C	-1.21	-2.94	29.26	0.0063
SB03028B1G10.f1	Gga.2542	TADA2L	Transcriptional adapter 2-alpha	-1.21	-2.84	29.26	0.0082
SB02032A1D01.f2	Gga.32515	FBXL3	F-box/LRR-repeat protein 3 (F-box and leucine-rich repeat protein 3A)	-1.20	-3.06	29.26	0.0047
SB03016A2D07.f1	Gga.39236	PLAA	Phospholipase A-2-activating protein	-1.20	-2.99	29.26	0.0056
SB03031A1D11.f1				-1.20	-2.90	29.26	0.0071
SB03001A1D08.f1	Gga.38335	SBF2	Myotubularin-related protein 13 (SET-binding factor 2)	-1.20	-2.86	29.26	0.0077
SB02038B2C05.f1	Gga.15381	RUFY3	RUN and FYVE domain containing 3	-1.20	-2.78	29.26	0.0095
SB02035B2E07.f1	Gga.43756	CRK	Proto-oncogene C-crk	-1.20	-2.96	29.26	0.0061
SB02038B1G09.f1	Gga.6283	LSM8	LSM8 homolog, U6 small nuclear RNA associated	-1.19	-2.88	29.26	0.0074
SB03017A1B05.f1	Gga.8083	ORMDL2	ORM1-like protein 2	-1.19	-2.80	29.26	0.0090
SB03018A2G04.f1	Gga.39790	ARF4	ADP-ribosylation factor 4	-1.19	-2.98	28.26	0.0058
SB02014B2G11.f1	Gga.9325	UBE2D2	Ubiquitin-conjugating enzyme E2 D2	-1.19	-3.06	29.26	0.0047
SB02034B1A04.f1	Gga.11467	SF3B4	Splicing factor 3B subunit 4 (Spliceosom	-1.19	-2.80	29.26	0.0091
SB03031B2E01.f1	Gga.10459	CCDC72	coiled-coil domain containing 72	-1.19	-2.90	27.26	0.0073
SB02034B1F09.f1	Gga.8044	PRDX1	Peroxiredoxin-1	-1.18	-3.04	29.26	0.0050
SB03030A1C02.f1	Gga.28933	FRMPD4	FERM and PDZ domain-containing protein 4	-1.18	-2.99	29.26	0.0056
SB03007B1C06.f1	Gga.4601	LIN7C	Lin-7 homolog C	-1.18	-3.53	29.26	0.0014
SB02011B1B11.f1				-1.17	-2.80	28.26	0.0091
SB03007B2G09.f1	Gga.36752	LRRC16A	Leucine-rich repeat-containing protein 16A	-1.17	-2.98	29.26	0.0058
SB02046A2B05.f1	Gga.5324	SYS1	Protein SYS1 homolog	-1.17	-3.47	29.26	0.0017
SB03004A1A11.f1	Gga.23549	AGXT2L1	Alanine--glyoxylate aminotransferase 2-like 1	-1.17	-3.08	29.26	0.0045
SB03042B2E06.f1	Gga.30094	BCLAF1	BCL2-associated transcription factor 1	-1.17	-2.84	29.26	0.0082
SB02010A2A07.f1	Hs.125713	FAM92B	family with sequence similarity 92, member B	-1.17	-3.02	29.26	0.0052
SB03029B2B12.f1	Gga.35990	ARID1B	AT-rich interactive domain-containing protein 1B	-1.17	-2.80	29.26	0.0090
SB02024B1B01.f1	Gga.5575	SEC61A2	Sec61 alpha 2 subunit	-1.17	-2.78	29.26	0.0094
SB02037A1E06.f1	Gga.4084	VDAC2	Voltage-dependent anion-selective channel protein 2	-1.17	-3.09	29.26	0.0044
SB03006A1B09.f1	Gga.25964	SPATA5	Spermatogenesis-associated protein 5	-1.16	-3.30	29.26	0.0025
SB03002B1A04.f1	Gga.27768	MRS2L	Magnesium transporter MRS2 homolog, mitochondrial Precursor	-1.16	-3.00	29.26	0.0055
SB02027A1C06.f1	Gga.28341	SLC25A22	solute carrier family 25 (mitochondrial carrier: glutamate), member 22	-1.16	-3.02	29.26	0.0052
SB03003B2G11.f1	Gga.9076	CCNC	Cyclin-C (hSRB11)	-1.16	-2.76	29.26	0.0099
SB02026A2C06.f1	Gga.8046	KRAS	GTPase KRas Precursor	-1.15	-2.83	29.26	0.0083
SB02025A2G06.f1	Gga.41380	RGPD2	similar to Ran-binding protein 2	-1.13	-2.78	29.26	0.0094
SB03043B2F11.f1	Gga.22011	CPSF6	Cleavage and polyadenylation specificity factor subunit 6	1.14	2.80	29.26	0.0091
SB02028A2H06.f1	Gga.4635	ST7L	Suppressor of tumorigenicity protein 7-like protein (ST7-related protein)	1.14	3.20	29.26	0.0033
SB03035A2F02.f1	Gga.16518	FAM49A	family with sequence similarity 49, member A	1.16	2.96	29.26	0.0060
SB02042B2A08.f1	Gga.34471	PHB2	Prohibitin-2	1.16	2.76	29.26	0.0100
SB03015A1A12.f1	Gga.3116	TRABD	TraB domain containing	1.16	2.92	29.26	0.0067
SB03018A1E05.f1	Gga.10045	VCP	valosin-containing protein	1.16	3.01	29.26	0.0054
SB03027B2B09.f1	Gga.39794	DASRAA	chromosomal passenger complex protein Dasra A	1.17	2.80	29.26	0.0089
SB03016B2A01.f1	Gga.34349	ZNF853-1	zinc finger protein 853	1.17	3.40	29.26	0.0020
SB02034B2E10.f1				1.17	2.90	29.26	0.0070
SB03014A2A08.f1.A	Gga.6335	PDCD10	Programmed cell death protein 10	1.17	3.20	29.26	0.0033
SB02031B2A12.f1	Gga.39326	KIAA0174	Uncharacterized protein KIAA0174	1.17	2.79	29.26	0.0093
SB02024B2A05.f1	Gga.15110	SFXN3	Sideroflexin-3	1.17	2.79	29.26	0.0091
SB03019A2F09.f1	Gga.12210	TTC1	Tetratricopeptide repeat protein 1	1.18	2.97	29.26	0.0059
SB02006B2B09.f1.B	Gga.3298	GCSH	Glycine cleavage system H protein	1.18	2.87	29.26	0.0076
SB020001000E06	Gga.1040	SLC2A1	Solute carrier family 2, facilitated glucose transporter member 1	1.18	3.22	29.26	0.0031
SB02007A1E06.f1.B				1.18	3.33	29.26	0.0023
SB020002000B02.M	Gga.11749	CDC42EP3	Cdc42 effector protein 3	1.18	2.85	29.26	0.0080
SB03023B2E01.f1.B	Gga.2627	NDUFAB1	Acyl carrier protein, mitochondrial Precursor	1.19	2.85	29.26	0.0079
SB010010000D12	Gga.11648	PHCA	Phytoceramidase, alkaline	1.19	2.76	29.26	0.0099
SB03046A2G02.f1	Gga.5664	PPP1CC	Protein phosphatase 1, catalytic subunit, gamma isoform	1.19	3.39	28.26	0.0021
SB03040B1E04.f1	Gga.30506	USP44	Ubiquitin carboxyl-terminal hydrolase 44	1.20	3.13	29.26	0.0040
SB03012A1D12.f1	Gga.28392	OTUD7B	OTU domain containing 7B	1.20	2.78	29.26	0.0094
SB02044A2A09.f1.B.M	Gga.643	SYT1	Synaptotagmin-1	1.20	3.01	29.26	0.0053
SB02026A1E01.f1.B.W	Gga.793	C11orf57	Uncharacterized protein C11orf57	1.20	3.30	29.26	0.0025
SB02021B1B09.f1	Gga.5681	PDCD7	Programmed cell death protein 7	1.20	2.94	29.26	0.0064
SB03040A1G07.f1	Gga.8157	SNRPD1	Small nuclear ribonucleoprotein Sm D1	1.20	2.92	29.26	0.0067
SB010004001C10	Gga.3302	CCT4	T-complex protein 1 subunit delta	1.20	3.21	29.26	0.0032
SB02024B1D12.f1	Gga.2333	C2orf25	Protein C2orf25, mitochondrial Precursor	1.20	2.83	29.26	0.0083
SB03026B1C11.f1	Gga.9751	MRPL14	39S ribosomal protein L14, mitochondrial Precursor	1.20	3.11	29.26	0.0041
SB02027A1E06.f1	Gga.3876	ATP6V1B2	V-type proton ATPase subunit B, brain isoform	1.21	2.99	29.26	0.0056
SB02032A1E03.f2	Gga.12304	C18orf55	chromosome 18 open reading frame 55	1.21	2.77	29.26	0.0097

SB03014A2F02.f1	Gga.12434	MAP3K7	Mitogen-activated protein kinase kinase kinase 7	1.21	2.81	29.26	0.0088
SB02044B2B03.f1	Gga.1218	LSM1	LSM1 homolog, U6 small nuclear RNA associated	1.21	3.04	29.26	0.0049
SB02047B1E07.f1	Gga.39036	BTF3	Transcription factor BTF3	1.21	2.97	28.26	0.0061
SB02009B1D09.f1	Gga.1454	LSM5	U6 snRNA-associated Sm-like protein LSm5	1.22	3.04	29.26	0.0049
SB03016B1C06.f1	Gga.6231	EIF2A	Eukaryotic translation initiation factor 2A	1.22	2.85	29.26	0.0080
SB010021000C12	Gga.4548	DDX5	DEAD (Asp-Glu-Ala-Asp) box polypeptide 5	1.22	3.24	29.26	0.0030
SB02037A1C11.f1	Gga.7507	ATP6V0D2	similar to Ac39/physophilin	1.22	2.87	29.26	0.0076
SB02016B1D09.f1	Gga.8976	ATG9A	Autophagy-related protein 9A (APG9-like 1)	1.22	3.10	29.26	0.0043
SB03041A1G03.f1	Gga.5904	COPS7A	COP9 signalosome complex subunit 7a	1.22	3.17	29.26	0.0036
SB02046B1D10.f1	Gga.17588	DIMT1L	Probable dimethyladenosine transferase	1.22	3.08	29.26	0.0045
SB03042B2D11.f1	Gga.2712	RALA	Ras-related protein Ral-A Precursor	1.22	3.73	29.26	0.0008
SB02015A1D07.f1	Gga.23864	CHKA	Choline kinase alpha (CK)	1.22	3.66	29.26	0.0010
SB03034A1G05.f1.B	Gga.41866	ZNF609	Zinc finger protein 609	1.22	2.76	29.26	0.0100
SB02014B2G05.f1				1.23	3.25	29.26	0.0029
SB010023000F01	Gga.11998	TPRKB	TP53RK-binding protein (PRPK-binding protein)	1.23	2.97	29.26	0.0059
SB02022B1B09.f1	Gga.27485	TTLL1	Probable tubulin polyglutamylase	1.23	2.81	29.26	0.0088
SB02047B1D10.f1	Gga.16654	NUP50	Nucleoporin 50 kDa	1.23	3.16	29.26	0.0036
SB03009A1A05.f1	Gga.14457	POLR1B	polymerase (RNA) I polypeptide B, 128kDa	1.23	3.22	29.26	0.0032
SB02024B2G08.f1	Gga.39572	AKR1B1	aldo-keto reductase family 1, member B1 (aldose reductase)	1.24	2.78	29.26	0.0094
SB02027B2F07.f1	Hs.66194	CTXN3	Cortexin-3 (Kidney and brain-expressed protein)	1.24	5.21	29.26	0.0000
SB03028B1B05.f1	Gga.11388	COQ3	coenzyme Q3 homolog, methyltransferase (S. cerevisiae)	1.24	2.77	29.26	0.0095
SB03017A2A07.f1	Gga.8959	NOL12	Nucleolar protein 12	1.24	3.25	29.26	0.0029
SB03047B1G02.f1	Gga.15212	CNTN3	Contactin-3 Precursor	1.24	3.01	29.26	0.0054
SB02047B1E10.f1	Hs.153521	KCNC4	Potassium voltage-gated channel subfamily C member 4	1.24	3.18	29.26	0.0035
SB03010B2G11.f1				1.24	2.88	29.26	0.0074
SB02040A1D10.f1	Hs.311100	C3orf75	chromosome 3 open reading frame 75	1.25	3.01	29.26	0.0053
SB03050A2F01.f1	Gga.22320	SARS	Seryl-tRNA synthetase, cytoplasmic	1.25	3.25	29.26	0.0029
SB03049B2E07.f1	Gga.20231	DNAJA1	DnaJ homolog subfamily A member 1	1.25	3.39	29.26	0.0020
SB03030B2G03.f1	Gga.42515	WDR33	WD repeat-containing protein 33	1.25	3.40	29.26	0.0020
SB02026A2G12.f1.B	Gga.21294	YIPF4	Protein YIPF4	1.25	2.81	29.26	0.0088
SB02034A1H07.f1				1.26	3.88	29.26	0.0005
SB03028B1H04.f1	Gga.8941	TXN2	Thioredoxin, mitochondrial Precursor	1.26	3.69	28.26	0.0010
SB02043A1H04.f1	Gga.13490	CKMT1A	Creatine kinase, ubiquitous mitochondrial Precursor	1.26	2.82	29.26	0.0086
SB03025B1D05.f2	Gga.22955	FKBP4	FK506-binding protein 4	1.27	3.33	29.26	0.0024
SB03017A1E11.f1	Gga.38332	ERGIC1	Endoplasmic reticulum-Golgi intermediate compartment protein 1	1.27	3.31	29.26	0.0025
SB03044B2E03.f1.B.M				1.27	3.00	29.26	0.0054
SB03030B2B10.f1	Gga.9582	RNPC3	RNA-binding protein 40	1.27	3.16	29.26	0.0036
SB02042A1A07.f1				1.27	3.09	29.26	0.0044
SB02024A2H03.f1	Gga.12108	MRP63	Ribosomal protein 63, mitochondrial	1.27	3.64	29.26	0.0010
SB03037B2D10.f1	Gga.4684	TMEM35	transmembrane protein 35	1.28	4.61	29.26	0.0001
SB03019B2F10.f1	Gga.36586	ADCK2	Uncharacterized aarF domain-containing protein kinase 2	1.28	2.78	29.26	0.0095
SB02036A2D08.f1	Gga.4219	HSPA5	Heat shock 70 kDa protein 5	1.28	3.28	29.26	0.0027
SB02022B2E12.f1	Gga.7435	OSBPL1A	Oxysterol-binding protein-related protein 1	1.28	3.18	29.26	0.0035
SB02040B2G02.f1	Gga.43613	B3GAT2	Galactosylgalactosylxylosylprotein 3-beta-glucuronosyltransferase 2	1.28	3.08	29.26	0.0045
SB02006A2E03.f1				1.29	2.86	29.26	0.0077
SB03025B1H05.f2	Gga.5702	GPR39	G protein-coupled receptor 39	1.29	3.89	29.26	0.0005
SB02023B1C02.f1	Gga.8074	TAF15	TATA box binding protein (TBP)-associated factor, 68kDa	1.29	3.07	29.26	0.0046
SB03044B1B10.f1	Gga.5900	DNAJA4	DnaJ homolog subfamily A member 4	1.29	4.05	29.26	0.0003
SB02020A1B09.f1	Gga.21673	ERMN	Ermin (Juxtanodin)	1.29	3.15	29.26	0.0038
SB010004001B01	Gga.9053	XBP1	X-box-binding protein 1 (XBP-1)	1.30	3.40	29.26	0.0020
SB02003B1B10.f1	Gga.8796	COPG	Coatomer subunit gamma	1.30	3.55	29.26	0.0013
SB03016B2A06.f1				1.30	3.13	29.26	0.0039
SB02044B2E10.f1	Gga.17356	SFRS8	Splicing factor, arginine/serine-rich 8	1.30	3.45	29.26	0.0017
SB010015000A11				1.30	2.96	29.26	0.0061
SB03036B1H10.f1	Gga.34734	NPTXR	Neuronal pentraxin receptor	1.30	4.86	29.26	0.0000
SB03021A1D05.f1	Gga.39100	LRWD1	Leucine-rich repeat and WD repeat-containing protein 1	1.31	3.00	29.26	0.0055
SB03049A1G10.f1	Gga.39684	ST6GALNAC2	Alpha-N-acetylgalactosaminide alpha-2,6-sialyltransferase 2	1.31	3.06	29.26	0.0047
SB03010B1F10.f1	Gga.3178	DACH2	Dachshund homolog 2	1.32	2.91	29.26	0.0068
SB02021A2H02.f1				1.32	3.42	29.26	0.0019
SB03029A2H05.f1	Gga.4678	SCCPDH	Probable saccharopine dehydrogenase	1.33	3.76	28.26	0.0008
SB02028A1G12.f1	Gga.1365	UBE2H	Ubiquitin-conjugating enzyme E2 H	1.33	2.79	29.26	0.0093
SB03046A2E03.f1	Gga.30265	INPP4A	Type I inositol-3,4-bisphosphate 4-phosphatase	1.34	2.83	29.26	0.0084
SB03030B1H12.f1				1.34	2.85	29.26	0.0078
SB03048B2F10.f1	Gga.9616	DCUN1D4	Defective in cullin neddylation protein 1-like protein 4	1.34	3.11	28.26	0.0043
SB03043B1D01.f1	Gga.12424	MGAM	maltase-glucoamylase (alpha-glucosidase)	1.34	3.16	29.26	0.0037
SB03041B1A02.f1.B	Gga.10026	VPS37B	Vacuolar protein sorting-associated protein 37B	1.34	4.03	29.26	0.0004
SB02029B2D06.f1	Gga.2155	ATP1A1	Sodium/potassium-transporting ATPase subunit alpha-1 Precursor	1.35	3.36	29.26	0.0022
SB03005B2B04.f1				1.35	2.98	29.26	0.0058
SB02005A2E12.f1	Gga.1946	C5orf42	Uncharacterized protein C5orf42	1.35	2.93	28.26	0.0066
SB02013B2B02.f1				1.36	3.11	29.26	0.0041
SB02012B2D07.f1	Gga.39236	PLAA	Phospholipase A-2-activating protein	1.36	3.70	29.26	0.0009
SB02037B1G04.f1	Gga.3869	PPP3CA	protein phosphatase 3 (formerly 2B), catalytic subunit, alpha isoform	1.36	2.98	29.26	0.0058
SB03004B1E12.f1	Gga.31942	MALT1-1	Mucosa-associated lymphoid tissue lymphoma translocation protein 1	1.37	2.87	29.26	0.0076
SB02016B1C02.f1	Gga.5145	CDV3	Protein CDV3 homolog	1.38	3.39	29.26	0.0020
SB03003A1G12.f1				1.38	2.96	29.26	0.0060
SB02020B1D09.f1				1.38	3.05	29.26	0.0048
SB03049A2C10.f1	Gga.13812	PDE1A	Calcium/calmodulin-dependent 3',5'-cyclic nucleotide phosphodiesterase 1A	1.39	2.87	29.26	0.0075
SB010022000B04	Gga.27568	TRAIP	TRAF-interacting protein (RING finger protein 206)	1.39	2.93	29.26	0.0065
SB03001A2H11.f1	Gga.17943	ROCK2	Rho-associated protein kinase 2	1.39	3.08	29.26	0.0045
SB02049B2D01.f1	Gga.7601	GALNS	N-acetylgalactosamine-6-sulfatase Precursor	1.40	3.30	29.26	0.0026
SB03005B2C11.f1.B	Gga.1208	DDX21	DEAD (Asp-Glu-Ala-Asp) box polypeptide 21	1.40	2.77	29.26	0.0096
SB03012A1G01.f1	Gga.10157	PROS1	protein S (alpha)	1.41	2.91	29.26	0.0068
SB02042B2C04.f1.B				1.41	2.76	29.26	0.0098
SB03013B2A01.f1	Gga.8300	DLAT	Dihydrolipoyllysine-residue acetyltransferase	1.41	2.85	29.26	0.0079
SB010007000E12	Gga.5334	RGS4	Regulator of G-protein signaling 4	1.41	3.54	27.26	0.0015
SB03021A1A12.f1				1.43	2.93	29.26	0.0066
SB02034A1G11.f1	Gga.4219	HSPA5	Heat shock 70 kDa protein 5	1.43	3.78	29.26	0.0007
SB03005B2F01.f1	Gga.5629	PLEKHG4	Pleckstrin homology domain-containing family G member 4	1.44	2.76	29.26	0.0100

SB02010B2A04.f1				1.47	2.82	28.26	0.0087
SB03030A2C10.f1				1.50	3.21	29.26	0.0032
SB03040B2E07.f1	Gga.822	ALDH4A1	aldehyde dehydrogenase 4 family, member A1	1.51	2.95	29.26	0.0062
SB02015B2H02.f1				1.53	3.57	29.26	0.0013
SB03049A2F11.f1	Gga.16800	MAPRE1	Microtubule-associated protein RP/EB family member 1	1.54	5.29	29.26	0.0000
SB02026A2G10.f1	Gga.1230	DNAJC12	DnaJ homolog subfamily C member 12	1.55	3.08	29.26	0.0044
SB03026A1B07.f1				1.57	3.48	29.26	0.0016
SB02030B1F03.f1				1.59	3.22	29.26	0.0031
SB02020B2E05.f1	Gga.28057	GTF2F2	general transcription factor IIF, polypeptide 2, 30kDa	1.67	4.69	29.26	0.0001
SB02018B2F10.f1	Gga.9380	PXK	PX domain-containing protein kinase-like protein	1.68	2.93	29.26	0.0065
SB03009B2H04.f1	Gga.5799	P4HA2	Prolyl 4-hydroxylase subunit alpha-2 Precursor	1.69	4.51	29.26	0.0001
SB03038B2D08.f1	Gga.40202	KNDC1	Protein very KIND	1.70	2.99	29.26	0.0056
SB02020A2A06.f1	Gga.24400	HACL1	2-hydroxyacyl-CoA lyase 1	1.71	2.96	29.26	0.0060
SB03044A2D03.f1	Hs.719230	HSPA2	Heat shock 70 kDa protein 2	1.75	4.42	29.26	0.0001
SB010007000B05.A				1.81	3.26	29.26	0.0028
SB03047B2G11.f1				1.85	3.21	29.26	0.0032
SB02044B1A07.f1	Gga.2902	HBA1	hemoglobin, alpha 1	1.92	3.02	29.26	0.0052
SB03046A2H10.f1				1.97	6.03	29.26	0.0000
SB03037B2H05.f1	Hs.719230	HSPA2	Heat shock 70 kDa protein 2	2.04	6.15	29.26	0.0000
SB02016A2B10.f1	Gga.45780	SLC6A5	solute carrier family 6 (neurotransmitter transporter, glycine), member 5	2.76	2.83	29.26	0.0083
SB02010B2A12.f1	Gga.10763	SLC6A4	Sodium-dependent serotonin transporter (5HT transporter)	3.29	2.85	29.26	0.0080
