# Supplementary material for: Seasonal Differences of Gene Expression Profiles in Song Sparrow (Melospiza melodia) Hypothalamus in Relation to Territorial Aggression
Source: PLoS One. 2009 Dec 4;4(12):e8182. doi: 10.1371/journal.pone.0008182 (PMC2780717; doi:10.1371/journal.pone.0008182)
Supplement: Table S3 — Complete list of cDNAs affected by the comparison SE vs. SC with cell-means model, p<0.01. The expressions in spring STI (SE) compared to spring control (SC) are shown in fold changes. (0.01 MB PDF) [file pone.0008182.s003.pdf]

Spot ID	UniGene ID	HGNC_symbol	Gene Description	Fold Change SE vs. SC	T Statistic	df	P-value
SB03032B1E02.f1	Gga.28126	SMARCAL1	SWI/SNF related matrix associated actin dependent regulator of chromatin 1	-5.65	-2.90	29.26	0.0071
SB03014B2H11.f1	Hs.284491	PDXK	Pyridoxal (pyridoxine, vitamin B6) kinase	-2.14	-3.08	29.26	0.0045
SB010007000B05.A	Gga.16601	NKAIN2	Na+/K+ transporting ATPase interacting 2	-1.76	-2.98	29.26	0.0058
SB02006A2D09.f1	Gga.39766	KIAA1609	KIAA1609	-1.61	-2.95	29.26	0.0062
SB03026A1G04.f1	Gga.23904	LAMA1	Laminin alpha-1 chain precursor	-1.47	-3.48	29.26	0.0016
SB02047A2H04.f1	Gga.39410	ZDHHC7	Zinc finger, DHHC-type containing 7	-1.45	-3.35	28.26	0.0023
SB03043B1D06.f1	Gga.33813	VLLH2748	Similar to VLLH2748	-1.37	-2.79	29.26	0.0091
SB02018B2H09.f1	Gga.4723	LECT1	Leukocyte cell derived chemotaxin 1	-1.35	-2.76	29.26	0.0099
SB02027A2G04.f1	Gga.5288	TEF	Thyrotroph embryonic factor	-1.33	-3.52	29.26	0.0014
SB02008A1D12.f1	Hs.404088	HAUS8	HAUS augmin-like complex, subunit 8	-1.33	-2.89	29.26	0.0073
SB02013B1E12.f1	Gga.11040	CCDC45	Coiled-coil domain containing 45	-1.31	-3.01	29.26	0.0054
SB02013B1C10.f1				-1.31	-2.76	29.26	0.0099
SB03025B1H05.f2	Hs.590869	NTSR1	Neurotensin receptor type 1	-1.29	-3.75	29.26	0.0008
SB02009A1H07.f1				-1.28	-3.24	29.26	0.0030
SB03032A2E11.f1	Gga.44093	TBCE	Tubulin-specific chaperone E	-1.28	-2.96	29.26	0.0060
SB02012A1G11.f1	Gga.4882	ASB7	Ankyrin repeat and SOCS box-containing 7	-1.27	-2.85	29.26	0.0079
SB02044B2E10.f1	Gga.17356	SFRS8	Splicing factor, arginine/serine-rich 8	-1.26	-2.95	29.26	0.0062
SB03029A1E07.f1.B	Hs.159234	FOXE1	Forkhead box E1	-1.24	-2.92	29.26	0.0066
SB02024B2A05.f1	Gga.15110	SFXN3	Sideroflexin-1	-1.23	-3.47	29.26	0.0017
SB03017A2A07.f1	Gga.8959	NOL12	Nucleolar protein 12	-1.22	-2.89	29.26	0.0071
SB03025B2A08.f2	Gga.22773	SETD1B	SET domain containing 1B	-1.22	-3.05	29.26	0.0048
SB02023A2E10.f1	Gga.4296	CLK3	CDC-like kinase 3	-1.21	-3.00	29.26	0.0055
SB03040A1E06.f1	Gga.16820	PHF13	PHD finger protein 13	-1.21	-3.08	29.26	0.0044
SB010002000B12	Gga.1342	KIF5B	Kinesin family member 5B	-1.21	-3.13	29.26	0.0040
SB03003A1E11.f1	Gga.17826	OXCT1	3-oxoacid CoA transferase 1	-1.20	-3.15	29.26	0.0037
SB03044B2F07.f1	Gga.18314	NSD1	Nuclear receptor binding SET domain protein 1	-1.20	-2.76	29.26	0.0099
SB03018B1E09.f1	Hs.517262	SON	SON DNA binding protein	-1.19	-2.88	29.26	0.0075
SB03009B1F02.f1	Gga.35033	RND3	Rho-related GTP-binding protein RhoE	-1.19	-3.26	29.26	0.0028
SB02041A1A03.f1	Gga.22563	KRR1	KRR1, small subunit (SSU) processome component	-1.18	-3.25	29.26	0.0029
SB02017B2G03.f1	Gga.39704	KLHL18	Kelch-like protein 18	-1.17	-2.76	29.26	0.0098
SB03051B1F05.f1.B.M				-1.15	-2.76	29.26	0.0098
SB02037B1G10.f1	Gga.39935	ATP5G3	ATP synthase, H+ transporting, mitochondrial F0 complex, subunit C3	1.14	2.78	29.26	0.0094
SB03005A1G05.f1	Gga.22077	LUC7L2	LUC7-like 2	1.15	2.85	29.26	0.0079
SB02034B1B11.f1	Gga.27810	RAB11FIP4	RAB11 family interacting protein 4 (class II)	1.15	2.79	29.26	0.0092
SB02022A2D12.f1	Gga.12569	FNBP1L	Formin binding protein 1-like	1.16	3.12	29.26	0.0040
SB03004A1A11.f1	Gga.23549	AGXT2L1	Alanine-glyoxylate aminotransferase 2-like 1	1.16	2.81	29.26	0.0087
SB03015B1G11.f1	Gga.3020	ACTR2	Actin-like protein 2	1.20	2.80	29.26	0.0089
SB02008A1D09.f1	Gga.35490	AAK1	AP2 associated kinase 1	1.20	3.16	29.26	0.0036
SB02038B1E05.f1				1.21	2.91	29.26	0.0068
SB02032B1D09.f2	Gga.1029	RBBP4	Retinoblastoma binding protein 4	1.21	3.10	29.26	0.0043
SB02028A1F12.f1				1.23	2.93	29.26	0.0065
SB03017A1B05.f1	Gga.8083	ORMDL1	ORM1-like protein 1	1.23	3.12	29.26	0.0040
SB010009001H01	Gga.3222	LIMK2	LIM domain kinase 2	1.23	2.99	29.26	0.0057
SB03034B1C11.f1.A				1.24	2.99	29.26	0.0056
SB03015B1E05.f1				1.24	3.10	29.26	0.0043
SB03013A1H09.f1	Gga.7961	C1orf128	chromosome 1 open reading frame 128	1.26	2.94	29.26	0.0064
SB02010A2C05.f1	Gga.8061	HSCB	Similar to J-type co-chaperone HSC20	1.26	3.29	29.26	0.0026
SB03029A2C01.f1	Gga.12450	GATAD1	GATA zinc finger domain containing 1	1.26	2.87	29.26	0.0076
SB03014B1B02.f1	Gga.30662	PEX13	Peroxisomal membrane protein PEX13	1.27	3.94	28.26	0.0005
SB02030A2A11.f1	Gga.2564	AP1S2	Adaptor-related protein complex 1, sigma 2 subunit	1.28	3.08	29.26	0.0045
SB03024B2A05.f1	Gga.9061	RTCD1	RNA 3-terminal phosphate cyclase	1.29	3.51	29.26	0.0015
SB03044B2B12.f1	Gga.11469	KCTD20	Potassium channel tetramerisation domain containing 20	1.29	3.37	29.26	0.0021
SB02028B2A07.f1	Gga.17250	CDKN1B	Cyclin-dependent kinase inhibitor 1B (p27, Kip1)	1.31	3.33	29.26	0.0024
SB02046B2A12.f1	Gga.4136	GPC5	Glypican-5 precursor	1.32	4.17	29.26	0.0002
SB03031A2G05.f1	Gga.15373	DDX31	Probable ATP-dependent RNA helicase DDX31	1.32	3.32	29.26	0.0024
SB02018A1F11.f1	Gga.34770	FAM120A	Family with sequence similarity 120A	1.33	2.77	29.26	0.0096
SB03032B2E02.f1	Gga.2852	ACVR2A	Activin receptor type 2 precursor	1.33	3.11	29.26	0.0042
SB03046B2A03.f1	Hs.370303	ZNF366	Zinc finger protein 366	1.36	3.09	29.26	0.0044
SB03050A1C01.f1	Gga.17065	CCDC66	Coiled-coil domain containing 66	1.39	2.92	29.26	0.0066
SB03002A2F12.f1	Gga.23549	AGXT2L1	Alanine-glyoxylate aminotransferase 2-like 1	1.48	3.83	29.26	0.0006
SB03041A2C02.f1	Gga.5899	FAM125B	Protein FAM125B	1.50	3.76	29.26	0.0008
SB03043B1E08.f1	Gga.2937	NFKBIA	NF-kappaB inhibitor alpha	1.52	3.58	29.26	0.0012
SB02049B2B06.f1	Hs.523012	DDIT4	DNA-damage-inducible transcript 4	1.56	3.53	29.26	0.0014
SB03036B1C02.f1	Gga.5101	ZBTB16	Zinc finger and BTB domain containing 16	1.71	5.63	29.26	0.0000
SB02027A2C02.f1	Gga.40320	GLP2R	Glucagon-like peptide 2 receptor Precursor	1.77	4.26	29.26	0.0002
SB03034B2D05.f1	Gga.5101	ZBTB16	Zinc finger and BTB domain containing 16	1.86	4.79	29.26	0.0000
SB02022A2D01.f1	Gga.11726	GADD45B	Growth arrest and DNA-damage-inducible, beta	2.16	5.16	29.26	0.0000
