# Supplementary material for: Seasonal Differences of Gene Expression Profiles in Song Sparrow (Melospiza melodia) Hypothalamus in Relation to Territorial Aggression
Source: PLoS One. 2009 Dec 4;4(12):e8182. doi: 10.1371/journal.pone.0008182 (PMC2780717; doi:10.1371/journal.pone.0008182)
Supplement: Table S4 — Complete list of cDNAs affected by the comparison AE vs. AC with cell-means model, p<0.01. The expressions in autumn STI (AE) compared to autumn control (AC) are shown in fold changes. (0.02 MB PDF) [file pone.0008182.s004.pdf]

Spot ID	Unigene_ID	HGNC_symbol	Gene Description	Fold Change AE vs. AC	T Statistics	df	P-value
SB010001001F02	Hs.439530	DYRK4	Dual-specificity tyrosine-(Y)-phosphorylation regulated kinase 4	-2.66	-2.88	29.26	0.0073
SB02025B1D06.f1	Gga.10284	VPS13C	Vacuolar protein sorting 13 homolog C	-2.42	-3.36	29.26	0.0022
SB02031B1D01.f1				-2.39	-2.91	29.26	0.0068
SB03011A2F09.f1	Hs.471779	LRRFIP1	Leucine rich repeat (in FLII) interacting protein 1	-2.19	-2.87	29.26	0.0075
SB03046A1H01.f1				-1.90	-2.97	29.26	0.0059
SB02039A2G02.f1				-1.89	-3.04	29.26	0.0050
SB03048B1D05.f1	Gga.32092	NPY1R	Neuropeptide Y receptor Y1	-1.88	-2.76	29.26	0.0098
SB03002A1B03.f1	Gga.19035	FAM26E	Family with sequence similarity 26, member E	-1.82	-2.81	29.26	0.0087
SB03046B1A02.f1.A	Gga.744	GATA3	GATA binding protein 3	-1.79	-2.81	29.26	0.0087
SB03017A2H04.f1	Gga.12177	IWS1	IWS1 homolog	-1.62	-2.90	29.26	0.0070
SB03035B1E06.f1	Hs.8595	MIIP	migration and invasion inhibitory protein	-1.51	-2.83	29.26	0.0084
SB03045B2F09.f1	Gga.40476	DGKH	Diacylglycerol kinase eta (DAG kinase eta)	-1.47	-2.91	29.26	0.0068
SB03038B2F02.f1	Gga.30037	CAND1	Cullin-associated and neddylation-dissociated 1	-1.46	-3.82	29.26	0.0006
SB02038A1G05.f1	Gga.19639	GPR133	G protein-coupled receptor 133	-1.46	-2.88	29.26	0.0075
SB03014B1H03.f1	Hs.592130	NR1D1	Nuclear receptor subfamily 1, group D, member 1	-1.42	-2.81	29.26	0.0087
SB02035B1A08.f1	Gga.4549	NONO	Non-POU domain containing, octamer-binding	-1.40	-3.80	29.26	0.0007
SB03028A2H11.f1	Gga.2827	TIMP3	TIMP metallopeptidase inhibitor 3	-1.39	-2.84	29.26	0.0081
SB02034B1H05.f1	Gga.15742	LARP4	La ribonucleoprotein domain family, member 4	-1.39	-3.25	28.26	0.0030
SB02003B2H08.f1	Gga.29968	ACOT11	Acyl-CoA thioesterase 11	-1.38	-3.06	29.26	0.0047
SB03029A1A05.f1				-1.38	-2.89	29.26	0.0072
SB02009B1H02.f1	Gga.14856	RPS6KA5	Ribosomal protein S6 kinase, 90kDa, polypeptide 5	-1.37	-3.10	29.26	0.0042
SB02013A2C04.f1	Gga.12457	C14orf159	Chromosome 14 open reading frame 159	-1.36	-3.37	29.26	0.0021
SB02007B2A12.f1	Gga.23166	HPS3	Hermansky-Pudlak syndrome 3	-1.34	-2.84	29.26	0.0081
SB03028A2B06.f1.A	Gga.22540	USP1	Ubiquitin specific peptidase 1	-1.34	-2.80	29.26	0.0089
SB02005A1C11.f1	Gga.26298	KLHL12	Kelch-like 12	-1.33	-2.99	29.26	0.0057
SB010022000E01				-1.33	-2.86	29.26	0.0077
SB03035A2C11.f1	Hs.370111	C2orf77	Chromosome 2 open reading frame 77	-1.32	-2.81	29.26	0.0087
SB03025B2H09.f2	Gga.31268	C3orf63	Chromosome 3 open reading frame 63	-1.32	-3.32	29.26	0.0024
SB02033B1F08.f1	Gga.11963	BLZF1	Basic leucine zipper nuclear factor 1 (JEM-1)	-1.31	-3.07	29.26	0.0046
SB03049B1F12.f1	Gga.1116	NEDD1	Neural precursor cell expressed, developmentally down-regulated 1	-1.31	-2.80	29.26	0.0091
SB03048A2G09.f1				-1.31	-2.81	29.26	0.0088
SB02027B1F01.f1	Gga.966	PTPRJ	Protein tyrosine phosphatase, receptor type, J	-1.30	-3.04	29.26	0.0049
SB03001B1G07.f1	Gga.5288	TEF	Thyrotrophic embryonic factor	-1.29	-3.94	29.26	0.0005
SB02023B2E08.f1	Gga.12008	GYG2	Glycogenin 2	-1.28	-2.76	29.26	0.0099
SB010011000C07	Gga.14840	C21orf66	Chromosome 21 open reading frame 66	-1.28	-2.90	29.26	0.0070
SB02048A1A09.f1	Gga.63	NEDD4L	Neural precursor cell expressed, developmentally down-regulated 4-like	-1.27	-2.83	29.26	0.0084
SB02047A2G08.f1	Gga.21092	PHF20L1	PHD finger protein 20-like 1	-1.27	-3.27	29.26	0.0027
SB03023A2C04.f1	Gga.26340	TRAK1	Trafficking protein, kinesin binding 1	-1.27	-3.01	29.26	0.0054
SB03009B1B05.f1	Gga.14586	SCN2A	Sodium channel, voltage-gated, type II, alpha subunit	-1.27	-2.83	29.26	0.0083
SB03044A2H07.f1	Gga.2720	CXCL14	Chemokine (C-X-C motif) ligand 14	-1.26	-3.04	29.26	0.0050
SB02010B1D10.f1	Gga.30233	RPS6KA2	Ribosomal protein S6 kinase, 90kDa, polypeptide 2	-1.26	-3.00	29.26	0.0054
SB02005B2D12.f1	Gga.3712	GAD2	Glutamate decarboxylase 2	-1.25	-2.92	29.26	0.0066
SB02024A2D05.f1				-1.25	-2.78	29.26	0.0094
SB02027B1H10.f1				-1.25	-3.04	29.26	0.0050
SB02013A1B04.f1	Hs.529272	MARCH7	Membrane-associated ring finger (C3HC4) 7	-1.25	-2.88	29.26	0.0073
SB03009A1H08.f1	Gga.2144	HSF2	Heat shock transcription factor 2	-1.23	-2.89	29.26	0.0072
SB03022B1E10.f1	Gga.30899	OTUD7A	OTU domain containing 7A	-1.23	-2.78	29.26	0.0094
SB03016A2E12.f1	Gga.29976	SYNE1	Spectrin repeat containing, nuclear envelope 1	-1.23	-2.80	29.26	0.0090
SB03018A2C06.f1				-1.22	-2.92	29.26	0.0066
SB03042A2B02.f1	Gga.30026	VANGL1	Vang-like 1 (van gogh, Drosophila)	-1.22	-3.06	29.26	0.0047
SB03031A1E09.f1	Hs.655657	EXOC6	Exocyst complex component 6	-1.22	-2.80	29.26	0.0089
SB02021A1H10.f1	Gga.37956	NOL8	Nucleolar protein 8	-1.21	-3.16	29.26	0.0036
SB03023A2B06.f1	Gga.1184	PSMB4	Proteasome subunit, beta type, 4	-1.21	-3.65	29.26	0.0010
SB02038B2G02.f1.B.E	Gga.2620	TTR	Transthyretin	-1.21	-2.78	29.26	0.0095
SB03002A2E10.f1	Gga.22662	PLTP	Phospholipid transfer protein	-1.21	-3.11	29.26	0.0041
SB03029B1E07.f1	Gga.42209	PDS5B	PDS5, regulator of cohesion maintenance, homolog B	-1.21	-2.95	29.26	0.0061
SB03038A1F02.f1	Gga.6024	PNRC1	Proline-rich nuclear receptor coactivator 1	-1.20	-2.92	29.26	0.0067
SB02031A1E06.f1	Gga.8788	SLC25A29	Solute carrier family 25, member 29	-1.20	-2.81	29.26	0.0089
SB03019B1G08.f1				-1.20	-2.91	29.26	0.0068
SB03012A2C05.f1	Gga.3128	THYN1	Thymocyte nuclear protein 1	-1.20	-3.14	28.26	0.0039
SB02010B2F06.f1				-1.20	-2.78	29.26	0.0094
SB02018B1A11.f1	Gga.9497	ZFYVE21	Zinc finger, FYVE domain containing 21	-1.20	-2.78	29.26	0.0094
SB02009B2F04.f1	Gga.5775	HNRNPA3	Heterogeneous nuclear ribonucleoprotein A3	-1.19	-3.88	29.26	0.0005
SB03021A1F06.f1	Gga.10406	CYLD	Cylindromatosis (turban tumor syndrome)	-1.19	-2.85	29.26	0.0080
SB02046B2D02.f1				-1.19	-2.78	29.26	0.0094
SB03017A2F11.f1	Gga.40014	CGGBP1	CGG triplet repeat binding protein 1	-1.18	-3.18	29.26	0.0035
SB02043A2E11.f1	Gga.34530	ATXN7	Ataxin 7-like 1	-1.18	-2.87	29.26	0.0076
SB02027A2G05.f1	Gga.28057	GTF2F2	General transcription factor IIF, polypeptide 2, 30kDa	-1.18	-3.45	29.26	0.0017
SB02022A2D12.f1	Gga.12569	FNBP1L	Formin binding protein 1-like	-1.17	-3.51	29.26	0.0015
SB010018000G02.B	Gga.17067	ALG9	Asparagine-linked glycosylation 9 homolog	-1.17	-2.78	29.26	0.0093
SB02025B1A04.f1	Gga.11465	DCUN1D5	Defective in cullin neddylation 1, domain containing 5	-1.17	-2.96	29.26	0.0060
SB02048B1H04.f1.A	Gga.21184	CDK2AP1	CDK2-associated protein 1	-1.17	-2.86	29.26	0.0077
SB02040A1C05.f1	Gga.21287	PSMD12	Proteasome 26S subunit, non-ATPase, 12	-1.16	-3.30	29.26	0.0025
SB03036B2G12.f1	Gga.41955	ACTR3	ARP3 actin-related protein 3 homolog	-1.16	-2.94	29.26	0.0063
SB03013A2H10.f1	Gga.12538	PSMD1	26S proteasome non-ATPase regulatory subunit 1	-1.16	-2.85	29.26	0.0078
SB03023B1F01.f1	Hs.517792	VHL	Von Hippel-Lindau syndrome	-1.16	-3.34	29.26	0.0023
SB02025A2G06.f1	Gga.41380	RGPD2	Similar to RanBP2 (Ran-binding protein 2)	-1.14	-2.97	29.26	0.0058
SB03008A1C01.f1	Hs.705605	TMEM110	Transmembrane protein 110	1.12	2.78	29.26	0.0094
SB02027B2F07.f1	Hs.66194	CTXN3	Cortexin 3	1.14	3.10	29.26	0.0043
SB03019B1B07.f1				1.14	3.27	29.26	0.0028
SB03028A2F06.f1	Gga.1080	MTHFD1	Methylenetetrahydrofolate dehydrogenase 1	1.15	3.03	29.26	0.0051
SB010002000G02	Gga.838	ATP12A	ATPase, H+/K+ transporting, nongastric, alpha polypeptide	1.15	2.85	29.26	0.0080
SB03031B2G07.f1	Gga.34829	EXOC4	Exocyst complex component 4	1.15	3.06	29.26	0.0048

SB02016A2E02.f1	Hs.119598	RPL3	Ribosomal protein L3 (RPL3)	1.15	2.90	29.26	0.0070
SB03025A1G04.f2	Gga.41022	IRS1	Insulin receptor substrate 1	1.15	2.91	29.26	0.0069
SB03035B2F07.f1	Hs.428214	MAML2	Mastermind like 2	1.16	2.84	29.26	0.0081
SB02028B2D07.f1.A	Gga.39472	H1FX	Similar to histone H1x	1.16	2.93	29.26	0.0065
SB02041A2A07.f1	Gga.40225	ADCYAP1R1	Adenylate cyclase activating polypeptide 1 (pituitary) receptor type I	1.16	2.84	29.26	0.0082
SB03045A1G01.f1	Gga.4205	RTN4	Reticulon 4	1.16	2.85	29.26	0.0080
SB020001000D12	Gga.37351	RNF2	Ring finger protein 2	1.17	2.76	29.26	0.0100
SB03044B2G04.f1	Gga.4613	APP	Amyloid beta (A4) precursor protein	1.17	3.08	29.26	0.0045
SB03035A2F02.f1	Gga.11550	FAM49A	Family with sequence similarity 49, member A	1.17	3.24	29.26	0.0030
SB03003B2G04.f1.A				1.18	2.98	29.26	0.0058
SB02029B2H06.f1	Gga.6309	TNK2	Tyrosine kinase, non-receptor, 2	1.18	2.88	29.26	0.0074
SB010021000E09	Gga.35108	NDRG4	NDRG family member 4	1.18	2.85	29.26	0.0079
SB02011B2D05.f1	Gga.8859	RNMTL1	RNA methyltransferase like 1	1.18	2.85	29.26	0.0079
SB03033A2B11.f1	Gga.10836	AKAP7	A kinase (PRKA) anchor protein 7	1.18	2.99	29.26	0.0057
SB02043B1C02.f1				1.19	2.76	29.26	0.0100
SB03024B2G08.f1	Gga.12043	PALMD	Palmdelphin	1.20	2.78	27.26	0.0098
SB02035A2G01.f1	Gga.30146	CEND1	Cell cycle exit and neuronal differentiation 1	1.20	2.78	29.26	0.0093
SB010020000G11	Hs.656271	SPG11	Spastic paraplegia 11	1.20	2.79	29.26	0.0092
SB02015A2H07.f1	Gga.35123	ANK2	Ankyrin 2, neuronal	1.20	2.77	29.26	0.0095
SB02022B1A07.f1	Gga.10425	VSNL1	Visinin-like 1	1.20	3.10	29.26	0.0042
SB02042A1G08.f1.B	Gga.10799	SFT2D2	SFT2 domain containing 2	1.21	2.83	29.26	0.0083
SB03046B1H08.f1				1.21	3.03	29.26	0.0051
SB03020B2F08.f1	Gga.42897	ENAH	Enabled homolog	1.21	2.86	29.26	0.0077
SB010023000F01	Gga.11998	TPRKB	TP53RK binding protein	1.21	2.79	29.26	0.0092
SB03038A2C12.f1	Gga.4833	PAPD1	PAP associated domain containing 1	1.21	3.48	29.26	0.0016
SB03004A1H02.f1	Gga.1345	YTHDC2	YTH domain containing 2	1.22	3.08	29.26	0.0045
SB03044B1B07.f1	Gga.21204	HSPA4L	Heat shock 70kDa protein 4-like	1.22	3.03	29.26	0.0050
SB03016A2D05.f1	Gga.33604	DUSP15	dual specificity phosphatase 15	1.22	4.13	29.26	0.0003
SB03032A1E07.f1	Gga.20111	PLEKHG1	Pleckstrin homology domain containing, family G (with RhoGef domain) me	1.22	2.86	29.26	0.0077
SB02023A2A01.f1	Gga.9753	MRPS17	Mitochondrial ribosomal protein S17	1.22	2.79	29.26	0.0091
SB02015A2E08.f1	Gga.16354	ATG4B	Autophagy related 4 homolog B	1.22	3.01	29.26	0.0053
SB02047B1G03.f1	Gga.4795	KLHDC2	Kelch domain containing 2	1.22	2.80	29.26	0.0090
SB03045A2E02.f1	Gga.22008	SUCLA2	Succinate-CoA ligase, ADP-forming, beta subunit	1.22	3.73	29.26	0.0008
SB02044B1C10.f1	Hs.9911	C12orf44	Chromosome 12 open reading frame 44	1.22	2.86	29.26	0.0077
SB03023B2E01.f1.B	Gga.2627	NDUFAB1	NADH dehydrogenase (ubiquinone) 1, alpha/beta subcomplex,1	1.22	3.32	29.26	0.0024
SB03013A2A05.f1	Gga.41623	CDKL5	Cyclin-dependent kinase-like 5	1.22	2.90	29.26	0.0070
SB03037B2G11.f1	Gga.6172	INPP5F	Inositol polyphosphate-5-phosphatase F	1.23	3.10	29.26	0.0042
SB02035B2A03.f1	Hs.113874	OMG	Oligodendrocyte-myelin glycoprotein	1.23	3.48	29.26	0.0016
SB010018000E11	Gga.2011	CLP1	Cleavage and polyadenylation factor I subunit, homolog	1.23	3.36	29.26	0.0022
SB03046B1B01.f1	Gga.12246	FEZ1	Fasciculation and elongation protein zeta 1	1.23	3.02	29.26	0.0052
SB02016B1D09.f1	Gga.8976	ATG9A	ATG9 autophagy related 9 homolog A	1.23	3.27	29.26	0.0028
SB03002A1G09.f1	Gga.21295	NUP85	Nucleoporin 85kDa	1.23	3.15	29.26	0.0038
SB02039A1D05.f1	Gga.1527	METTL7A	Methyltransferase like 7A	1.24	3.01	29.26	0.0053
SB02049A2D10.f1	Gga.6269	FARSB	Phenylalanyl-tRNA synthetase, beta subunit	1.24	3.09	29.26	0.0043
SB02011A1C10.f1	Hs.301654	KIAA1024	KIAA1024	1.25	2.83	29.26	0.0084
SB02027B2E05.f1	Gga.2942	LAMP2	Lysosomal-associated membrane protein 2	1.25	3.62	29.26	0.0011
SB02028A1D07.f1				1.25	3.72	29.26	0.0009
SB02048A2C02.f1	Gga.7650	GSK3B	Glycogen synthase kinase 3 beta	1.25	3.53	29.26	0.0014
SB02030B1B09.f1	Gga.38006	PLXDC2	Plexin domain containing 2	1.25	2.77	29.26	0.0096
SB03040A2C03.f1	Hs.705605	TMEM110	Transmembrane protein 110	1.26	2.89	29.26	0.0072
SB02044B2C12.f1	Gga.23874	RASGRF1	RAS protein-specific guanine nucleotide-releasing factor 1	1.26	3.15	29.26	0.0038
SB03024A1B09.f1.A	Gga.1327	C9orf119	Chromosome 9 open reading frame 119	1.26	2.87	29.26	0.0075
SB02030B2D07.f1	Gga.12977	DDB1	Damage-specific DNA binding protein 1, 127kDa	1.27	3.34	29.26	0.0023
SB03002B2B03.f1	Gga.4833	PAPD1	PAP associated domain containing 1	1.27	2.93	29.26	0.0066
SB03003A1D04.f1	Gga.18371	LARP6	La ribonucleoprotein domain family, member 6	1.27	2.80	29.26	0.0089
SB02035B1E09.f1	Gga.22322	STXBP3	Syntaxin binding protein 3	1.27	3.28	29.26	0.0027
SB03022A2C10.f1	Hs.2178	HIST2H2BE	Histone cluster 2, H2be	1.27	2.97	29.26	0.0059
SB03031A2C12.f1	Gga.16427	USP14	Ubiquitin carboxyl-terminal hydrolase 14	1.27	3.13	29.26	0.0039
SB02020A2F10.f1	Gga.37123	VWA1	Von Willebrand factor A domain containing 1	1.28	3.39	29.26	0.0020
SB02012A2H03.f1	Gga.3003	PRKRIP1	PRKR interacting protein 1	1.29	3.68	29.26	0.0009
SB02031B2D01.f1	Hs.501857	DENND5A	DENN/MADD domain containing 5A	1.30	3.30	29.26	0.0025
SB02038B2E05.f1	Hs.658489	CLCC1	Chloride channel CLIC-like protein 1 Precursor	1.30	3.34	29.26	0.0023
SB03047A1D09.f1	Gga.28074	MUTYH	MutY homolog	1.31	3.79	29.26	0.0007
SB02037A1B10.f1	Gga.22887	NR2F6	Nuclear receptor subfamily 2, group F, member 6	1.31	3.20	29.26	0.0033
SB02040A1H09.f1				1.31	3.05	29.26	0.0048
SB03037A1F03.f1	Gga.39722	B3GALNT2	Beta-1,3-N-acetylgalactosaminyltransferase 2	1.32	3.50	29.26	0.0015
SB02006B2F02.f1	Gga.2240	FREQ	Frequenin homolog	1.33	3.07	29.26	0.0046
SB03023A1F08.f1	Hs.651849	FAM38B2	Family with sequence similarity 38, member B2	1.34	2.98	29.26	0.0057
SB02008B1D10.f1	Gga.1509	PRPF3	PRP3 pre-mRNA processing factor 3 homolog	1.34	2.83	29.26	0.0084
SB02029B2D06.f1	Gga.2155	ATP1A1	ATPase, Na+/K+ transporting, alpha 1 polypeptide	1.34	3.31	29.26	0.0025
SB03044A2E05.f1	Gga.20682	CORO2A	Coronin, actin binding protein, 2A	1.35	3.52	29.26	0.0014
SB03007A1F01.f1	Hs.369104	C3orf57	Similar to ADMP	1.36	3.10	29.26	0.0043
SB02013B1A09.f1	Gga.44170	PTTG1IP	Pituitary tumor-transforming 1 interacting protein	1.36	3.71	29.26	0.0009
SB03043B1D01.f1	Gga.12424	KIF21A	Kinesin family member 21A	1.37	3.39	29.26	0.0020
SB03035A2F09.f1	Gga.17167	GABRA1	Gamma-aminobutyric acid (GABA) A receptor, alpha 1	1.39	2.98	29.26	0.0057
SB02006A2B03.f1	Gga.21290	WDR70	WD repeat domain 70	1.39	2.90	29.26	0.0071
SB02013B1F07.f1	Gga.2421	DIP2B	Disco-interacting protein 2 homolog B	1.39	3.05	28.26	0.0050
SB03046A2B05.f1	Hs.501857	DENND5A	DENN/MADD domain containing 5A	1.39	3.19	29.26	0.0034
SB02018B2C06.f1	Gga.22154	PAQR8	Progestin and adipoQ receptor family member VIII	1.41	3.50	29.26	0.0015
SB03049A1D01.f1	Gga.25612	POLG2	Polymerase, gamma 2, accessory subunit	1.42	2.88	29.26	0.0074
SB03024B1C04.f1	Gga.20183	IARS	Isoleucyl-tRNA synthetase	1.48	2.87	29.26	0.0076
SB02008A1E12.f1	Gga.28050	LOC777232	Similar to MGC81165 protein	1.49	3.30	29.26	0.0026
SB03006A2H08.f1	Gga.42149	ATRN	Attractin	1.49	2.80	29.26	0.0090
SB03026A1B07.f1	Gga.7850	ABCB10	ATP-binding cassette, sub-family B (MDR/TAP), member 10	1.51	3.20	29.26	0.0033
SB03025B2G12.f2	Gga.43069	ALDH4A1	Aldehyde dehydrogenase 4 family, member A1	1.52	2.90	29.26	0.0071

SB02036B1D07.f1				1.71	3.18	29.26	0.0035
SB03042A2E11.f1				1.72	2.98	29.26	0.0057
SB03045B1C07.f1				1.78	3.09	29.26	0.0044
SB03039B1G08.f1.A	Gga.5250	HDHD3	Haloacid dehalogenase-like hydrolase domain containing 3	1.87	2.76	29.26	0.0099
SB03002A2H02.f1				2.28	2.89	29.26	0.0072
