# Supplementary material for: Seasonal Differences of Gene Expression Profiles in Song Sparrow (Melospiza melodia) Hypothalamus in Relation to Territorial Aggression
Source: PLoS One. 2009 Dec 4;4(12):e8182. doi: 10.1371/journal.pone.0008182 (PMC2780717; doi:10.1371/journal.pone.0008182)
Supplement: Table S5 — Complete list of cDNAs affected by the interaction (season×social) with cell-means model, p<0.01. The expressions in spring STI (SE) compared to spring control (SC) and autumn STI (AE) compared to autumn control (AC) are shown in fold changes. (0.01 MB PDF) [file pone.0008182.s005.pdf]

Spot ID	Unigene_ID	HGNC_symbol	Gene Description	Fold Change SE vs.SC	Fold Change AE vs. AC	T statistics	df	P-value
SB02008A1D12.f1	Hs.404088	HAUS8	HAUS augmin-like complex, subunit 8	-1.33	1.13	3.00	29.26	0.0054
SB02032B1D07.f2	Gga.6044	FAM175A	Family with sequence similarity 175, member A	-1.33	1.34	3.39	29.26	0.0020
SB03026A1B07.f1	Gga.7850	ABCB10	ATP-binding cassette, sub-family B, member 10	-1.30	1.51	3.60	29.26	0.0012
SB03025B1H05.f2	Hs.590869	NTSR1	Neurotensin receptor 1	-1.29	1.14	4.11	29.26	0.0003
SB03032B2E06.f1	Gga.11322	STXBP1	Syntaxin binding protein 1	-1.27	1.13	2.77	29.26	0.0097
SB03032B2A09.f1	Gga.43154	EHBP1	EH domain binding protein 1	-1.24	1.17	3.07	29.26	0.0046
SB02034A2D11.f1.A	Gga.29328	TP53BP2	Tumor protein p53 binding protein, 2	-1.24	1.52	2.76	29.26	0.0098
SB03029B1A06.f1				-1.24	1.09	2.76	29.26	0.0099
SB02016B1C02.f1	Gga.5145	CDV3	CDV3 homolog	-1.22	1.26	3.13	29.26	0.0040
SB02023A2E10.f1	Gga.4296	CLK3	Dual specificity protein kinase CLK3	-1.21	1.06	2.78	29.26	0.0094
SB03004A1H08.f1	Gga.1225	CCT6A	Chaperonin containing TCP1, subunit 6A	-1.21	1.14	3.33	29.26	0.0024
SB02030A2H08.f1	Gga.24273	CDH13	Cadherin-13 precursor	-1.21	1.33	3.13	29.26	0.0039
SB02006A2E03.f1	Gga.25128	COX5B	Cytochrome c oxidase polypeptide Vb (VI)	-1.21	1.22	3.06	29.26	0.0047
SB02041B2A07.f1				-1.20	1.24	3.10	29.26	0.0043
SB03003A1E11.f1	Gga.17826	OXCT1	3-oxoacid CoA transferase 1	-1.20	1.08	3.24	29.26	0.0030
SB02041A1A03.f1	Gga.22563	KRR1	KRR1, small subunit (SSU) processome component	-1.18	1.13	4.03	29.26	0.0004
SB02048A2E09.f1	Hs.506989	RFC5	Replication factor C (activator 1) 5	-1.18	1.20	2.93	29.26	0.0066
SB03013A2A07.f1				-1.18	1.14	3.07	29.26	0.0046
SB02018B2E03.f1.A	Gga.5189	TTC19	Tetratricopeptide repeat protein 19	-1.18	1.11	2.85	29.26	0.0080
SB03002A1G08.f1	Gga.37302	TMEM45B	Transmembrane protein 45a	-1.17	1.21	2.98	29.26	0.0058
SB02035B2A03.f1	Hs.113874	OMG	Oligodendrocyte myelin glycoprotein	-1.16	1.23	4.16	29.26	0.0003
SB03036B1H10.f1	Hs.91622	NPTXR	Neuronal pentraxin receptor	-1.16	1.12	3.28	29.26	0.0027
SB02014A1G07.f2	Gga.33766	MAN2A2	Alpha-mannosidase Iix	-1.16	1.26	2.90	29.26	0.0071
SB02020A1A04.f1	Gga.22901	PRC1	Protein regulator of cytokinesis 1	-1.16	1.11	2.77	29.26	0.0097
SB02014B1A01.f1	Gga.2375	CTNNA2	Catenin (cadherin-associated protein), alpha 2	-1.15	1.16	2.97	29.26	0.0059
SB03022A2B10.f1	Gga.32082	IRS1	Insulin receptor substrate 2 (IRS-2)	-1.15	1.09	3.13	29.26	0.0039
SB03035A2C12.f1	Gga.30037	CAND1	Cullin-associated and neddylation-dissociated 1	-1.15	1.28	2.80	29.26	0.0090
SB03020A2D12.f1	Gga.39562	TBC1D20	TBC1 domain family, member 20	-1.14	1.19	2.80	29.26	0.0090
SB02008B1H12.f1	Hs.670146	CACNG2	Calcium channel, voltage-dependent, gamma subunit 2	-1.14	1.24	2.87	29.26	0.0075
SB03004A1H02.f1	Gga.1345	YTHDC2	YTH domain containing 2	-1.14	1.22	3.55	29.26	0.0013
SB03041B1A02.f1.A	Gga.10026	VPS37B	Vacuolar protein sorting 37 homolog B	-1.14	1.14	2.91	29.26	0.0069
SB03013A2E02.f1	Gga.229	HINT1	Histidine triad nucleotide binding protein 1	-1.12	1.09	2.76	29.26	0.0099
SB03043B1D01.f1	Gga.12424	KIF21A	Kinesin family member 21A	-1.12	1.37	3.16	29.26	0.0036
SB03038B1B08.f1	Gga.2489	SLC39A9	Solute carrier family 39 (zinc transporter), member 9	-1.11	1.11	2.91	29.26	0.0069
SB02029B2D06.f1	Gga.2155	ATP1A1	ATPase, Na+/K+ transporting, alpha 1 polypeptide	-1.11	1.34	3.08	29.26	0.0045
SB02016B1D09.f1	Gga.8976	ATG9A	ATG9 autophagy related 9 homolog A	-1.10	1.23	3.33	29.26	0.0024
SB03013B2G07.f1	Gga.5818	MRPL51	Mitochondrial ribosomal protein L51	-1.10	1.12	2.78	29.26	0.0094
SB020001000D12	Gga.37351	RNF2	Ring finger protein 2	-1.10	1.17	3.09	29.26	0.0043
SB02043B1C02.f1				-1.09	1.19	2.83	29.26	0.0083
SB02031B2D01.f1	Hs.501857	DENND5A	DENN/MADD domain containing 5A	-1.09	1.30	3.03	29.26	0.0051
SB03035A2F02.f1	Gga.11550	FAM49A	Family with sequence similarity 49, member A	-1.07	1.17	3.23	29.26	0.0031
SB03002A1G09.f1	Gga.21295	NUP85	Nucleoporin 85kDa	-1.06	1.23	2.78	29.26	0.0094
SB03023A2B06.f1	Gga.1184	PSMB4	Proteasome subunit beta type 4 precursor	1.05	-1.21	-3.21	29.26	0.0032
SB02040A1C05.f1	Gga.21287	PSMD12	Proteasome 26S subunit, non-ATPase, 12	1.06	-1.16	-3.13	29.26	0.0040
SB03002A2E10.f1	Gga.22662	PLTP	Phospholipid transfer protein precursor	1.06	-1.21	-2.85	29.26	0.0079
SB02009B2F04.f1	Gga.5775	HNRNPA3	Heterogeneous nuclear ribonucleoprotein	1.07	-1.19	-3.70	29.26	0.0009
SB02016B1C07.f1	Gga.9325	UBE2T	Ubiquitin-conjugating enzyme E2 T	1.08	-1.18	-2.79	29.26	0.0091
SB03033B1H08.f1	Gga.28477	PAPD5	PAP associated domain containing 5	1.11	-1.18	-2.90	29.26	0.0071
SB02031A1E06.f1	Gga.8788	SLC25A29	Solute carrier family 25, member 29	1.11	-1.20	-3.02	29.26	0.0052
SB02016B2D05.f1				1.12	-1.10	-2.78	29.26	0.0094
SB02012A1E02.f1	Hs.375837	C10orf93	Putative uncharacterized protein C10orf93	1.12	-1.17	-2.80	29.26	0.0090
SB02027A2B05.f1	Gga.15424	WDSOF1	WD repeats and SOF1 domain containing	1.13	-1.16	-2.76	29.26	0.0098
SB03018A2G04.f1	Gga.39790	ARF5	ADP-ribosylation factor 4	1.15	-1.11	-2.83	28.26	0.0085
SB02049A1B07.f1	Gga.16420	N4BP1	Nedd4 binding protein 1	1.16	-1.24	-2.85	29.26	0.0080
SB010003000H07	Gga.16842	SPCS1	Signal peptidase complex subunit 1	1.16	-1.10	-2.90	29.26	0.0070
SB02022A2D12.f1	Gga.12569	FNBP1L	Formin binding protein 1-like	1.16	-1.17	-4.68	29.26	0.0001
SB03048A2C01.f1	Gga.5407	TCEA2	Transcription elongation factor A (SII), 2	1.18	-1.25	-3.00	29.26	0.0054
SB03028B1D02.f1	Gga.42380	AMFR	Autocrine motility factor receptor	1.18	-1.11	-3.20	29.26	0.0033
SB03031B2E01.f1	Gga.10459	CCDC72	coiled-coil domain containing 72	1.19	-1.08	-2.85	27.26	0.0082
SB02026B1E10.f1	Gga.22421	PDZD11	PDZ domain containing protein 11	1.20	-1.12	-2.85	29.26	0.0079
SB02038B1E05.f1				1.21	-1.07	-2.88	29.26	0.0075
SB03017A1B05.f1	Gga.5902	ORMDL2	ORM1-like protein 2 (Adoplin-2)	1.23	-1.07	-2.97	29.26	0.0059
SB010009001H01	Gga.3222	LIMK2	LIM domain kinase 2	1.23	-1.12	-3.36	29.26	0.0022
SB02036B1C11.f1	Gga.34693	PION	Protein pigeon homolog	1.23	-1.13	-2.92	29.26	0.0066
SB03034B1C11.f1.A				1.24	-1.06	-2.76	29.26	0.0100
SB03015B1E05.f1				1.24	-1.09	-3.16	29.26	0.0037
SB03046A2A02.f1	Gga.21371	NIT2	Nitrilase family, member 2	1.26	-1.48	-2.91	29.26	0.0068
SB03014B1B02.f1	Gga.30662	PEX13	Peroxisome biogenesis factor 13	1.27	-1.10	-4.03	28.26	0.0004
SB03048A2G09.f1				1.27	-1.31	-3.70	29.26	0.0009
SB02046B2A12.f1	Gga.4136	Gpc5	Glypican-5 precursor	1.32	1.01	-2.92	29.26	0.0067
SB03031A2G05.f1	Gga.15373	DDX31	Probable ATP-dependent RNA helicase DDX3	1.32	-1.20	-3.98	29.26	0.0004
SB03032B2E02.f1	Gga.2852	ACVR2A	Activin receptor type 2 precursor	1.33	-1.07	-2.77	29.26	0.0096
SB03048B2D08.f1.A	Gga.8180	HMGN3	High mobility group nucleosomal binding domain 3	1.33	-1.25	-2.86	29.26	0.0077
SB03046B2A03.f1	Hs.370303	ZNF366	Zinc finger protein 366	1.36	-1.08	-2.77	29.26	0.0095
SB03024B1A12.f1	Gga.32314	SLC38A4	Solute carrier family 38, member 4	1.37	-1.21	-2.81	29.26	0.0087
SB02014A2B08.f2				1.39	-1.16	-2.87	29.26	0.0076
SB03006A1B07.f1	Gga.944	CENPH	Centromere protein H	1.39	-1.40	-3.23	29.26	0.0030
SB03002A2F12.f1	Gga.23549	AGXT2L1	Alanine-glyoxylate aminotransferase 2-like 1	1.48	1.00	-2.76	29.26	0.0099
SB03046A2G09.f1				1.67	-1.68	-2.92	29.26	0.0067
SB03036B1C02.f1	Gga.5101	ZBTB16	Zinc finger and BTB domain containing 16	1.71	1.14	-3.06	29.26	0.0047
SB02032A2C07.f2	Gga.30876	DMAP1	DNA methyltransferase 1 associated protein 1	1.75	-1.41	-2.82	29.26	0.0085
SB02027A2C02.f1	Gga.40320	GLP2R	Glucagon-like peptide 2 receptor Precursor	1.77	-1.10	-3.58	29.26	0.0012

SB03034B2D05.f1	Gga.5101	ZBTB16	Zinc finger and BTB domain containing 16	1.86	1.00	-3.45	29.26	0.0017
SB02022A2D01.f1	Gga.11726	GADD45B	Growth arrest and DNA-damage-inducible, beta	2.16	1.16	-3.02	29.26	0.0053
SB03038B1F04.f1	Gga.2620	TTR	Transthyretin precursor	5.41	-2.96	-2.83	29.26	0.0083
SB02032B2F01.f2	Gga.2620	TTR	Transthyretin precursor	5.90	-2.88	-2.79	29.26	0.0092
SB02026B2H07.f1	Gga.2620	TTR	Transthyretin precursor	6.32	-2.84	-2.78	29.26	0.0093
SB02015A2G08.f1	Gga.2620	TTR	Transthyretin precursor	6.95	-3.55	-2.97	29.26	0.0058
